# A Non‐Canonical Role for Hepatocyte MLKL in Promoting Mitochondrial Dysfunction and Senescence in the Aging Liver

**DOI:** 10.1111/acel.70618

**Published:** 2026-07-03

**Authors:** Sabira Mohammed, Chao Jiang, Travis Pennington, Shylesh Bhaskaran, Phoebe Ohene‐Marfo, Kara F. Kneuper, Constantin Georgescu, Kamille Pitts, Albert Tran, Zongkai Peng, Amit Singh, Zhibo Yang, Tiangang Li, Bethany Hannafon, Courtney Houchen, Jonathan D. Wren, Andriy Yabluchanskiy, Veronica Galvan, Nagib Ahsan, Michael Kinter, Tommy L. Lewis, Sathyaseelan S. Deepa

**Affiliations:** ^1^ Stephenson Cancer Center University of Oklahoma Health Campus Oklahoma City Oklahoma USA; ^2^ Department of Biochemistry & Physiology University of Oklahoma Health Campus Oklahoma City Oklahoma USA; ^3^ Aging & Metabolism Research Program Oklahoma Medical Research Foundation Oklahoma City Oklahoma USA; ^4^ Genes and Human Disease Research Program Oklahoma Medical Research Foundation Oklahoma City Oklahoma USA; ^5^ Division of Digestive Diseases and Nutrition, Department of Medicine University of Oklahoma Health Campus Oklahoma City Oklahoma USA; ^6^ Department of Chemistry and Biochemistry The University of Oklahoma Norman Oklahoma USA; ^7^ Harold Hamm Diabetes Center University of Oklahoma Health Campus Oklahoma City Oklahoma USA; ^8^ Department of Obstetrics and Gynecology University of Oklahoma Health Campus Oklahoma City Oklahoma USA; ^9^ Department of Neurosurgery University of Oklahoma Health Campus Oklahoma City Oklahoma USA; ^10^ Oklahoma Center for Geroscience & Healthy Brain Aging University of Oklahoma Health Campus Oklahoma City Oklahoma USA; ^11^ Mass Spectrometry, Proteomics and Metabolomics Core Facility, Stephenson Life Sciences Research Center The University of Oklahoma Norman Oklahoma USA

**Keywords:** aging, fission‐fusion, hepatocyte, liver, mitochondria, MLKL, oxidative stress, senescence

## Abstract

Liver aging is characterized by chronic inflammation and metabolic dysfunction that drive progression of metabolic dysfunction‐associated steatotic liver disease (MASLD). Necroptosis, a pro‐inflammatory form of cell death via the Receptor‐Interacting serine/threonine‐Protein Kinase 1 (RIPK1)‐RIPK3‐Mixed Lineage kinase domain Like pseudokinase (MLKL) pathway, is activated in aging livers, and systemic inhibition of this pathway reduces hepatic inflammation and pathology. The cell type‐specific role of necroptosis in liver aging, however, is unclear. Notably, RIPK3 is suppressed in hepatocytes under metabolic disease, suggesting necroptosis independent functions for MLKL. Here, we show that MLKL is elevated in aged hepatocytes and drives liver aging via a non‐necroptotic mechanism. Using hepatocyte‐specific MLKL‐overexpressing mice (MLKL^HepOE^), we find that MLKL overexpression does not induce necroptosis but instead promotes cellular senescence, evidenced by increased p16INK4a and p21WAF1/Cip1 and elevated senescence associated secretory phenotype (SASP). Mechanistically, MLKL induces hepatocyte mitochondrial dysfunction, with impaired respiration, altered mitochondrial dynamics, and increased reactive oxygen species, implicating oxidative stress as a contributing mechanism. This mitochondrial stress is associated with enhanced release of pro‐inflammatory extracellular vesicles (EVs) and induction of senescence in hepatocytes and non‐parenchymal cells. While hepatocytes contribute substantially to total senescent burden by abundance, macrophages emerge as a senescence‐enriched population, indicating amplification of senescence through non‐cell‐autonomous signaling. Collectively, these findings reveal a non‐lethal, non‐necroptotic function of hepatocyte MLKL in promoting liver inflammaging via mitochondrial dysfunction and paracrine senescence signaling, identifying MLKL as a regulator of hepatic aging and a potential therapeutic target in age‐associated liver disease.

## Graphical abstract

Aging‐associated MLKL upregulation in hepatocytes induces mitochondrial dysfunction and oxidative stress, accompanied by increased release of pro‐inflammatory extracellular vesicles. These changes are associated with increased p16^+^/p21^+^ senescence in hepatocytes and non‐parenchymal cells (macrophages, stellate cells, and endothelial cells), suggesting a potential paracrine mechanism that amplifies SASP signaling and liver inflammation.

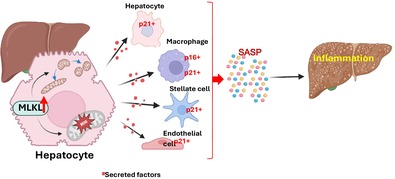

## 1 Introduction

Aging is characterized by a progressive decline in cellular function, metabolic dysregulation, and the development of a chronic, low‐grade inflammatory state termed inflammaging. This persistent inflammation is a central hallmark of aging and is strongly associated with the onset and progression of age‐related diseases, including neurodegenerative, cardiovascular, and metabolic disorders. Multiple mechanisms contribute to inflammaging, including cellular and immune senescence, alterations in gut microbiota, coagulation abnormalities, and the chronic release of damage‐associated molecular patterns (DAMPs) (Franceschi and Campisi 2014).

Necroptosis, a regulated and highly pro‐inflammatory form of programmed cell death, represents a major source of DAMPs release. This pathway is commonly triggered by inflammatory stimuli such as tumor necrosis factor‐α (TNFα) and is mediated by RIPK1 and RIPK3, culminating in activation of the terminal effector mixed lineage kinase domain‐like pseudokinase (MLKL) via phosphorylation and subsequent oligomerization. Oligomerized MLKL translocates to cellular membranes, leading to membrane disruption and release of DAMPs (e.g., HMGB1, ATP, S100 proteins). These DAMPs activate toll‐like receptors (TLRs) and NF‐κB signaling in innate immune cells, amplifying inflammation and creating a feed‐forward loop that further promotes necroptosis. Activation of necroptosis is reported in several age‐associated diseases, including neurodegenerative, cardiovascular, chronic kidney, and liver diseases such as MASLD (Royce et al. 2019).

Liver aging is characterized by increased hepatic inflammation, elevated circulating and intrahepatic cytokines, and susceptibility to chronic liver diseases, such as MASLD (Stahl et al. 2020). The prevalence of MASLD rises markedly with age, and the resulting chronic inflammatory environment promotes disease progression to cirrhosis and hepatocellular carcinoma, underscoring the need to define mechanisms that drive liver inflammaging (He et al. 2024). DAMPs released from injured hepatocytes have been implicated in chronic liver inflammation and fibrogenesis (Shan et al. 2024), and necroptosis has emerged as a prominent mode of hepatic cell death in MASLD and metabolic dysfunction‐associated steatohepatitis (MASH) under pathological conditions. In mouse models, genetic or pharmacological inhibition of RIPK1, RIPK3, or MLKL attenuates hepatic inflammation and fibrosis, supporting a causative role for necroptosis in liver pathology (Gautheron et al. 2014; Majdi et al. 2020; Wu et al. 2020).

We previously demonstrated that markers of necroptosis (phosphorylated MLKL and MLKL oligomerization) are elevated in the livers of *Sod1* ^*−/−*^ mice, a model of accelerated aging, and that inhibition of RIPK1 with necrostatin‐1 s markedly reduced hepatic inflammation and fibrosis (Mohammed, Nicklas, et al. 2021). Necroptosis markers similarly increase with age in wild‐type mice and correlate with liver inflammation, steatosis, and fibrosis, while global genetic (*Ripk3* ^*−/−*^, *Mlkl* ^*−/−*^) or pharmacological inhibition (necrostatin‐1 s) of necroptosis significantly attenuates these age‐associated pathologies (Mohammed et al. 2025; Mohammed, Thadathil, et al. 2021). However, because these approaches rely on whole‐body inhibition, the cell type‐specific contribution of necroptosis signaling to liver inflammaging remains undefined. This limitation is particularly relevant given recent reports showing that RIPK3 is epigenetically silenced in hepatocytes in metabolic diseases, including MASLD (Preston et al. 2022), questioning whether canonical necroptosis can occur in this cell type in aging.

To address this knowledge gap, we generated a hepatocyte‐specific MLKL overexpression mouse model (MLKL^HepOE^). Our rationale for overexpressing MLKL was three‐fold: (1) it is the terminal effector of necroptosis; (2) overexpression of the N‐terminal domain of MLKL is sufficient to induce cell death even without necroptotic stimuli or RIPK3 in HEK293T cells (Dondelinger et al. 2014); and (3) MLKL protein is significantly upregulated (2–3 fold) in aging livers and hepatocytes, and in pathological conditions, correlating with inflammation and fibrosis (Mohammed et al. 2025; Mohammed, Thadathil, et al. 2021; Ohene‐Marfo et al. 2024). Our data show that hepatocyte MLKL overexpression did not induce necroptosis, but instead caused mitochondrial dysfunction, oxidative stress, and the activation of a multicellular senescence program in the liver. These findings identify a non‐necroptotic function of hepatocyte MLKL in promoting hepatic senescence and liver inflammaging.

## 2 Methods

The detailed methodology is provided in the supplementary section.

### 2.1 Experimental Animals

All procedures were approved by the OU Health Campus IACUC. Male C57BL/6J mice were housed under standard conditions (20°C ± 2°C, 12 h light/dark cycle) and fed NIH‐31 chow. Young (7 months) and old (24 months) mice were obtained from NIA aged rodent colonies.

### 2.2 Generation of Rosa26‐MLKL and MLKL^HepOE^ Mice

A conditional Rosa26‐MLKL knock‐in mouse was generated by ViewSolid Biotech (Oklahoma, USA) by inserting Mlkl cDNA (Mlkl 202, Dharmacon/Horizon Discovery) into the Rosa26 locus on a C57BL/6J background using CRISPR/Cas9‐mediated homology‐directed repair (Figure S1A). Transgene expression is Cre‐dependent, enabled by excision of a loxP‐flanked STOP cassette in a tissue‐specific manner. Hepatocyte‐specific MLKL overexpression (MLKL^HepOE^) was achieved by tail vein injection of AAV8‐TBG‐Cre (2 × 10^11^ GC/mouse; VB1724, Vector Biolabs), while control mice received AAV8‐TBG‐Null (70600‐Pre) at the same dose.

### 2.3 Primary Hepatocyte Isolation and Treatment

Primary hepatocytes were isolated from mice using the Liver Perfusion Kit (130‐128‐030; Miltenyi Biotec, CA, USA) according to the manufacturer's protocol. The non‐parenchymal cell (NPC) fraction was collected from the supernatant after hepatocyte pelleting. Hepatocytes were treated with the necroptosis‐inducing cocktail TBZ (TNFα, BV6, and zVAD) for 6 h (Hoff et al. 2023), and TBZ‐treated RAW 264.7 macrophages served as a positive control (Hao et al. 2021).

### 2.4 Western Blotting

Western Blotting was performed as previously described (Mohammed et al. 2025).

### 2.5 Detection of 4‐Hydroxynonenal (4‐HNE) Adducts

4‐Hydroxynonenal (4‐HNE) adducts were detected by western blotting as described previously (Mohammed, Nicklas, et al. 2021).

### 2.6 Immunofluorescence Staining

Immunofluorescence staining was done as previously described (Mohammed, Thadathil, et al. 2021).

### 2.7 Quantitative Real‐Time PCR

Quantitative real‐time PCR (qPCR) was performed as described previously (Mohammed, Thadathil, et al. 2021). Table S1 lists the primers used for the study.

### 2.8 Histological Analysis of Liver Sections

Formalin‐fixed and paraffin‐embedded liver tissue sections were H&E‐stained (Stephenson Cancer Center core) and imaged on an ECHO REVOLVE R4 microscope (Discover Echo Inc); three random non‐overlapping fields per sample were analyzed.

### 2.9 Liver Triglyceride Quantification

Liver triglyceride quantification was done using a triglyceride colorimetric assay kit (#10010303, Cayman Chemical Company) as we have described (Mohammed et al. 2025).

### 2.10 SPiDER‐βGal Assay

SPiDER‐βGal assay was performed using SPiDER‐βGal assay kit (#SG02‐10, Dojindo). Images were taken with Leica SP8 confocal microscope at 630 magnification, 4 random non‐overlapping fields per sample were recorded.

### 2.11 Transmission Electron Microscopy

Transmission electron microscopy was performed at the Oklahoma Medical Research Foundation Imaging Facility as previously described (Bhaskaran et al. 2018). Mitochondrial area was quantified from five sections per mouse using ImageJ.

### 2.12 TUNEL Assay

TUNEL assay was performed using the DeadEnd Fluorometric TUNEL System (#G3250, Promega) and images were taken with Leica SP8 confocal microscope at 200× magnification, 4 random non‐overlapping fields per sample were analyzed for TUNEL positive cells.

### 2.13 ELISA for Alanine Transaminase (ALT) and Aspartate Aminotransferase (AST)

ELISA for Alanine Transaminase (ALT) and Aspartate Aminotransferase (AST) was performed using ALT or AST colorimetric activity assay kits (#700260, or #701640, Cayman Chemical Company, MI, USA).

### 2.14 Human Plasma MLKL

Human plasma MLKL was quantified using MLKL ELISA Kit (#MBS9300811, MyBioSource). Sample details are provided Tables S2 and S3, and Supporting Information.

### 2.15 Label Free Quantitative Proteomic Analysis

A total of 100 μg of liver lysate was used in the analysis as described previously (Mohammed et al. 2025). Proteomics data can be found in the MassIVE database via MSV000097694.

### 2.16 Targeted Mitochondrial Proteomics

Quantitative targeted proteomics was performed to assess changes in mitochondrial enzyme levels in liver tissue, as previously described (Ohene‐Marfo et al. 2024).

### 2.17 Untargeted Lipidomics Analysis

Untargeted lipidomics was performed by Creative Proteomics (Shirley, NY, USA) using UPLC‐MS to profile liver lipid composition.

### 2.18 Overexpression of MLKL in AML12 Cells

AML12 cells (#CRL2254, ATCC) were transfected with cDNA (pcDNA3.1 + C‐DYK, GenScript) or pcDNA‐MLKL‐Flag (ORF Clone, #OMU41768D, GenScript) using Lipofectamine reagent (#L3000008, Invitrogen). Experiments were performed 24 h post transfection.

### 2.19 Cell Viability Assay

Cell viability was measured using Cell Counting kit‐8 (96992, Sigma‐Aldrich).

### 2.20 Measurement of Mitochondrial Respiration and Oxidative Stress

Mitochondrial respiration was measured using the Agilent Seahorse XF96 Extracellular Flux Analyzer (Mito Stress Test) in AML12 cells transfected with pcDNA or MLKL‐Flag and treated with vehicle or 250 nM MitoQ (18 h). Oxidative stress was assessed by MitoSOX Red staining and imaged on a Nikon Ti Eclipse inverted microscope.

### 2.21 Live‐Cell Imaging Using Photoactivatable GFP and LAMP1/Mt‐mScarlet Reporters

AML12 cells were transfected with DDK tagged MLKL (pCAG MLKL‐DDK) and fluorescent reporter (pCAG 2xmt‐paGFP p2a 2xmt‐mScarlet, pCAG 2xmt‐mScarlet or pCAG Lamp1‐mEmerald). Following overexpression, live time‐lapse imaging of the cells was done. For photo‐activation experiments, 5 by 5 μm ROIs were selected in each cell imaged. For analysis of mitochondrial dynamics, a square ROI of 5 by 5 μm was used, and ROI intensity statistics were quantified across the time course. Data was plotted over the entire time course, with comparisons made with end points and slopes.

### 2.22 Exosome Isolation and Nanoparticle Tracking Analysis

AML12 cells were transfected with pcDNA or pcDNA‐MLKL‐FLAG; HepG2 cells were transfected with either siControl or siMLKL. Exosome isolation was performed using the Total exosome isolation kit (# 4478359, Invitrogen) and analyzed using NanoSight for particle size and concentration analysis.

### 2.23 RNA In Situ Hybridization (RNAscope)

Paraffin embedded liver samples from control and MLKL^HepOE^ mice were sectioned freshly and stained. All reagents were ordered from Advanced Cell Diagnostics (ACD) and performed according to the user manual (Wang et al. 2012). Probes for p16, p21, Albumin, Agdre1, Desmin, and Pecam1 were used. Imaging was performed with Axioscan Zeiss Slide Scanner and analyzed with HALO Image Analysis Platform version 3.6.4134 (Indica Labs Inc.) through the FISH module. The percentage of p16 or p21 cells in a certain cell type was calculated as p16 or p21 positive cell numbers [cell type]/Number of [cell type] × 100%.

### 2.24 Bioinformatics

For untargeted proteomics, heatmaps, and volcano plots were generated by SRplot (https://www.bioinformatics.com.cn/en), a free online platform for data analysis and visualization. All pathway analyzes were performed using ShinyGO 0.80 bioinformatics platform (Ge et al. 2020).

### 2.25 Statistical Analyzes

All data are represented as mean ± SEM. Two‐tailed unpaired *t*‐test or one‐way or two‐way ANOVA was used to analyze data with GraphPad Prism. *p* < 0.05 is considered statistically significant.

## 3 Results

### 3.1 Generation and Characterization of MLKL^HepOE^ Mouse Model

**FIGURE 1:**
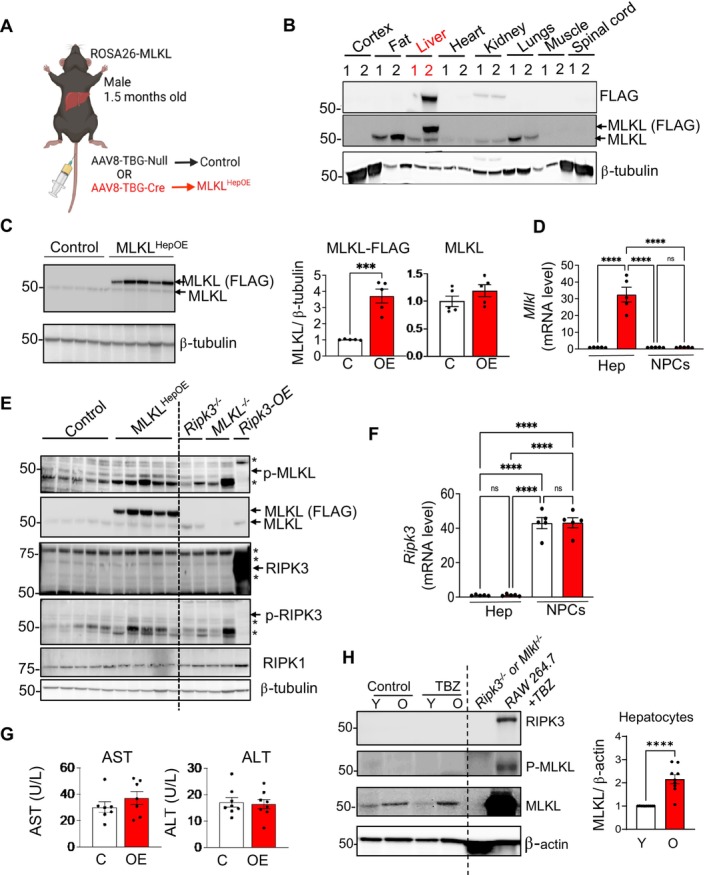
Hepatocyte‐specific MLKL overexpression does not induce necroptosis or liver injury. (A) Experimental schematic for hepatocyte‐specific MLKL overexpression. (B) Western blot showing FLAG, MLKL (exogenous and endogenous), and β‐tubulin across tissues in control (1) and MLKL^HepOE^ (2) mice. (C) *Left*: Liver MLKL and β‐tubulin expression in control *(C)* and MLKL^HepOE^ *(OE)* mice at 4 months post‐injection. *Right*: Quantification of MLKL (FLAG and endogenous) normalized to β‐tubulin. (D) qPCR of *Mlkl* mRNA in hepatocytes (Hep) and non‐parenchymal cells (NPCs). (E) Western blot of liver lysates for p‐MLKL, MLKL, p‐RIPK3, RIPK3, and RIPK1. β‐tubulin served as loading control. *Ripk3* ^*−/−*^, *Mlkl* ^*−/−*^, and Ripk3‐FLAG‐transfected HepG2 lysates were used as controls. (F) qPCR of *Ripk3* mRNA in Hep and NPCs. (G) Plasma AST and ALT levels. (H) *Left*: Primary hepatocytes from young (Y) and old (O) mice treated with TBZ (TNFα, BV6, zVAD‐fmk; 6 h) and analyzed for RIPK3, p‐MLKL, and MLKL with appropriate controls [RIPK3 blot: Negative (*Ripk3* ^*−/−*^ whole liver lysate); positive (RAW264.7 cells+TBZ); p‐MLKL and MLKL blot: Negative (*Mlkl* ^*−/−*^ whole liver lysate); positive (RAW264.7 cells+TBZ)]. *Right*: Quantification of MLKL normalized to β‐actin. Data are mean ± SEM; *n* = 5/group (C–F), *n* = 7–8/group (G, H). Control (white), MLKL^HepOE^ (red). Statistics: Two‐tailed unpaired *t*‐test (C, G, H) or one‐way ANOVA (D, F). *p < 0.05, **p < 0.01, ***p < 0.001, ****p < 0.0001; ns, not significant.

To generate hepatocyte‐specific MLKL‐overexpressing mice, 1.5‐month‐old ROSA26‐MLKL mice were injected via tail vein with AAV8‐TBG‐Cre. Control mice received AAV8‐TBG‐Null virus (Figure 1A). Efficient MLKL overexpression was detected two weeks post‐injection (Figure S1B). Mice were analyzed 4 months later (~5.5 months old) to capture effects of sustained MLKL overexpression. Body weight was similar between groups (Figure S1C), but MLKL^HepOE^ mice showed increased absolute liver weight, while liver‐to‐body weight and other tissue weight ratios were unchanged (Figure S1D,E). Western blotting across multiple tissues confirmed that MLKL overexpression was confined to the liver (Figure 1B). Hepatic *Mlkl* transcript levels increased 9‐fold in MLKL^HepOE^ mice, with MLKL‐FLAG expression elevated 3.5‐fold and endogenous MLKL levels unchanged (Figures S1F and 1C). Hepatocyte and non‐parenchymal cell (NPC) isolation confirmed that *Mlkl* overexpression was restricted to hepatocytes (Figures 1D and S1G).

### 3.2 Hepatocyte‐Specific MLKL Overexpression Does Not Induce Necroptosis, Apoptosis, or Liver Injury

To investigate the effect of hepatocyte MLKL overexpression on necroptosis, we performed western blot analysis of necroptosis pathway proteins in liver tissues from control and MLKL^HepOE^ mice. Phosphorylated MLKL (p‐MLKL), the necroptosis marker, was not detected, as similar bands were present in *Mlkl* ^*−/−*^ liver tissues (Figure 1E). Similarly, phosphorylated RIPK3 (p‐RIPK3) showed non‐specific bands in both groups, confirmed using *Ripk3* ^*−/−*^ liver tissues. RIPK3 protein was undetectable in liver tissue from young mice, validated with RIPK3‐FLAG overexpressing HepG2 lysates as positive control (Figure 1E). Analysis of *Ripk3* transcript levels in hepatocyte and non‐parenchymal cell (NPC) fractions from control and MLKL^HepOE^ liver tissues showed that *Ripk3* expression was negligible in hepatocytes, but detectable in NPCs (Figure 1F). RIPK1 levels were similar between groups (Figure 1E). Although MLKL overexpression did not alter necroptosis pathway proteins, MLKL oligomer formation increased 2‐fold in MLKL^HepOE^ mice (Figure S1H). Hepatocyte injury assessment by measuring plasma levels of ALT and AST showed no significant differences between MLKL^HepOE^ and control mice (Figure 1G).

We next assessed RIPK3 and MLKL expression in hepatocytes isolated from young (7‐month‐old) and aged (24‐month‐old) mice. RIPK3 protein was undetectable in hepatocytes from either age group, whereas MLKL expression was increased 2‐fold in hepatocytes from aged mice compared with young mice (Figure 1H). Consistent with RIPK3 being undetectable, treatment with the necroptosis inducer TBZ failed to induce MLKL phosphorylation in primary hepatocytes (Figure 1H). In contrast, RAW 264.7 macrophages, used as a positive control, expressed RIPK3 and showed robust MLKL phosphorylation in response to TBZ (Figure 1H, S1I).

To assess whether MLKL overexpression induces cell death through alternative pathways such as apoptosis, we performed TUNEL staining. The analysis revealed comparable levels of TUNEL‐positive cells in liver tissues from both control and MLKL^HepOE^ mice (Figure S1J). This was further corroborated by measuring expression of markers of apoptosis, cleaved caspase‐3 and cleaved PARP, in the liver tissues of control and MLKL^HepOE^ mice, which showed similar levels of expression (Figure S1K). Thus, hepatocyte‐specific MLKL overexpression did not induce necroptosis, apoptosis, or hepatocyte injury, despite increased MLKL oligomer formation, suggesting a non‐lethal role for hepatocyte MLKL.

### 3.3 Impact of Hepatocyte MLKL Overexpression on the Liver Proteome

**FIGURE 2:**
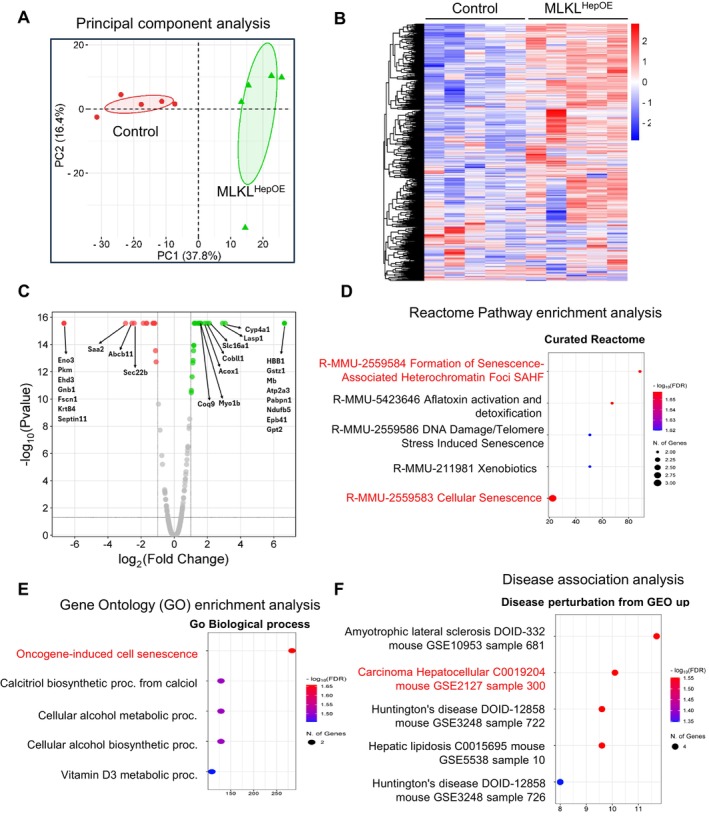
Untargeted label‐free quantitative proteomics of control and MLKL^HepOE^ livers. (A) Principal component analysis showing distinct clustering of control (red) and MLKL^HepOE^ (green) liver proteomes (*n* = 5/group). (B) Heatmap of hierarchical clustering of all quantified proteins with Z‐score–normalized expression. (C) Volcano plot of differentially expressed proteins (±1.5‐fold, *p* < 0.05); upregulated (green) and downregulated (red) proteins are indicated, with selected hits labeled. (D) Reactome pathway enrichment of upregulated proteins. (E) Gene Ontology biological process enrichment of differentially expressed proteins. (F) Disease association analysis of differentially expressed proteins. In (D–F), dot size represents the number of associated genes, and color indicates statistical significance (red, higher; blue, lower).

To assess the consequence of hepatocyte MLKL overexpression, we performed untargeted label free quantitative proteomics on livers from control and MLKL^HepOE^ mice. Principal component analysis revealed (PCA) clear separation between groups, with tightly clustered controls and increased variability among MLKL^HepOE^ samples (Figure 2A), and heatmap analysis demonstrated distinct proteomic signatures (Figure 2B). Differential expression analysis identified upregulated proteins (CYP4A1, LASP1, SLC16A1, COBLL1, ACOX1, COQ9, MYO1B) linked to fatty acid metabolism, mitochondrial function, and oxidative stress, and downregulated proteins (SAA2, ABCB11, SEC22B, ENO3, PKM, SEPTIN11) associated with lipid transport, glycolysis, and cytoskeletal organization (Figure 2C; Table S4).

Pathway enrichment analyzes revealed significant enrichment of cellular senescence related pathways, including senescence‐associated heterochromatin foci (SAHF), with cellular senescence showing the strongest association (Figure 2D). Gene Ontology analysis further highlighted oncogene induced senescence (Figure 2E), while disease‐associated pathway analysis revealed enrichment of pathways linked to hepatocellular carcinoma and hepatic lipidosis (Figure 2F). Together, these data suggest that hepatocyte MLKL overexpression promotes senescence‐associated proteomic remodeling and alters metabolic and disease‐related pathways in the aging liver.

### 3.4 Hepatocyte MLKL Overexpression Induces Multicellular Cellular Senescence in the Liver

**FIGURE 3:**
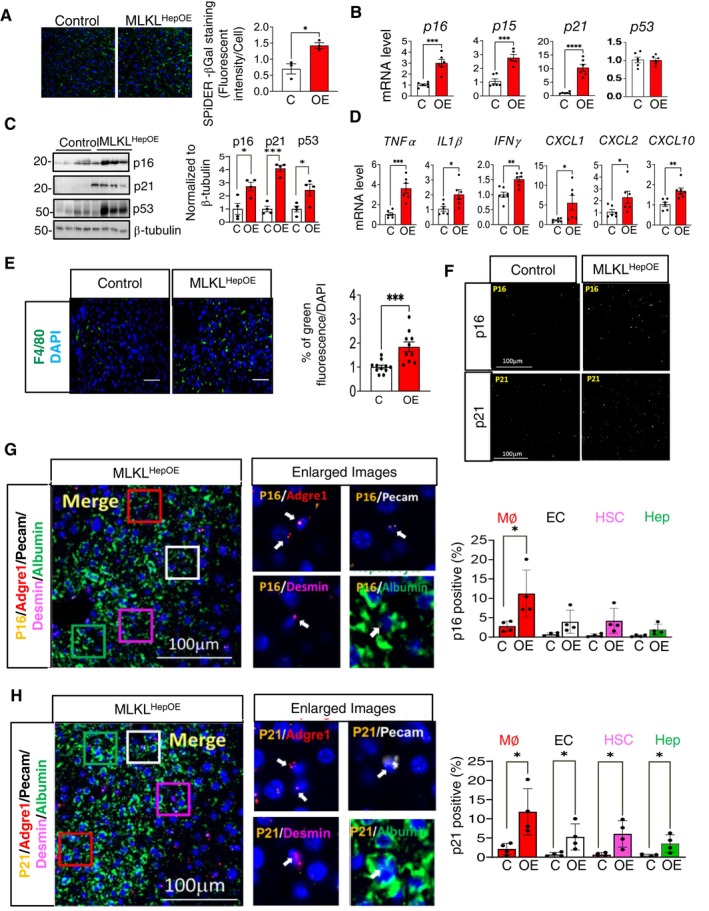
Hepatocyte MLKL overexpression induces cellular senescence, inflammatory macrophage recruitment, and multicellular senescence. (A) Left: SPiDER‐βGal staining of liver sections from control *(C)* and MLKL^HepOE^ *(OE)* mice (green), with DAPI (blue). Magnification: 630×. *Right*: Quantification of β‐gal‐positive signal per cell (*n* = 3/group). (B) qPCR of cell cycle arrest markers (p16, p15, p21, p53) in liver. (C) *Left*: Western blot for p16, p21, p53, and β‐tubulin. *Right*: Quantification normalized to β‐tubulin. (D) qPCR of SASP cytokines and chemokines. (E) *Left*: Immunofluorescence of F4/80 (green) with DAPI (blue). Scale bar: 50 μm; 200×. *Right*: Quantification normalized to DAPI. (F) RNAscope detection of p16 and p21 in liver sections. Scale bar: 100 μm. (G,H) Co‐localization of p16 (G) or p21 (H) with cell‐type markers: Adgre1 (macrophages, MΦ), Pecam1 (endothelial cells, EC), desmin (stellate cells, HSC), and albumin (hepatocytes, Hep). Scale bar: 100 μm. *Right*: Percentage of p16‐ or p21‐positive cells within each cell type. Data are mean ± SEM; *n* = 4–5/group (B–H), Control (white), MLKL^HepOE^ (red). Statistics: Two‐tailed unpaired *t*‐test. *p < 0.05, **p < 0.01, ***p < 0.001, ****p < 0.0001.

To evaluate hepatic senescence, we performed SPiDER‐β‐gal staining to detect senescence‐associated β‐galactosidase activity. MLKL^HepOE^ livers displayed significantly increased SA‐β‐Gal positivity (Figure 3A). Additionally, senescence‐associated cell‐cycle inhibitors *p16* (3‐fold), *p15* (2.7‐fold), and *p21* (10‐fold) were upregulated at the mRNA level, while *p53* transcripts were unchanged (Figure 3B). At the protein level, p16 (2.7‐fold), p21 (4‐fold), and p53 (2.5‐fold) were significantly increased in MLKL^HepOE^ livers (Figure 3C). SASP cytokines and chemokines, *TNFα* (3.6‐fold), *IL1β* (1.7‐fold), *IFN*γ (1.5‐fold), *CXCL1* (5.5‐fold), *CXCL2* (2.3‐fold), and *CXCL10* (1.7‐fold), were elevated (Figure 3D), whereas SASP growth factors and proteases were unchanged (Figure S2A). Consistent with a pro‐inflammatory SASP, MLKL^HepOE^ livers showed increased macrophage recruitment, with a 2‐fold rise in F4/80^+^ cells (Figure 3E) and elevated M1 markers (*CD68*: 1.8‐fold; *CD11c*: 2‐fold; *TLR4*: 1.5‐fold) (Figure S2B). M2 markers were unchanged (Fizz1, CD206) or modestly increased (Arg1: 1.2‐fold; CD163: 2.2‐fold) (Figure S2C).

To define the cellular distribution and spatial localization of senescent cells within the liver, we performed RNA‐scope in situ hybridization to quantify and map cell type‐specific senescence. Consistent with increased p16 and p21 transcripts in MLKL‐overexpressing livers (Figure 3B), RNAscope confirmed their elevation (Figure 3F). Quantification of p16 and p21 positivity across liver cell types, normalized to the total number of each cell type, revealed a similar distribution, with macrophages (Adgre1^+^) exhibiting the highest proportion of positive cells (12%), hepatocytes (Albumin^+^) showing lower positivity (2%–5%), and stellate (Desmin^+^) and endothelial cells (Pecam^+^) displaying intermediate levels (4%–5%). Notably, p21 positivity was significantly increased across all cell populations, whereas p16 showed a significant increase only in macrophages (Figure 3G,H). Detailed quantification of cell type‐specific senescence is provided in Figure S2D–H. Cell type abundance analysis (relative distribution of liver cell populations) showed an expansion of non‐parenchymal cells in MLKL‐overexpressing livers (Figure S2D). Absolute p16 or p21 double‐positive cell counts demonstrated a broad increase across both hepatocytes and non‐parenchymal cells (Figure S2E,F). Transcript intensity (RNAscope puncta per cell, reflecting expression levels within individual senescent cells) showed enhanced p16 or p21 expression (Figure S2G,H), while normalization to copies per 100 cells indicated an overall increase in senescence burden, with macrophages exhibiting the highest enrichment (Figure S2I,J). Collectively, although hepatocytes exhibited a lower proportion of p16^+^ and p21^+^ cells compared to macrophages, their high abundance in the liver suggests that hepatocytes represent a substantial fraction of the total senescent cell pool. In contrast, macrophages, despite being less abundant, showed the highest enrichment of p16 and p21 positivity, indicating increased susceptibility to senescence.

### 3.5 MLKL Overexpression Induces Mitochondrial Dysfunction and Alters Lipid Metabolism and Promotes Steatosis in the Liver

**FIGURE 4:**
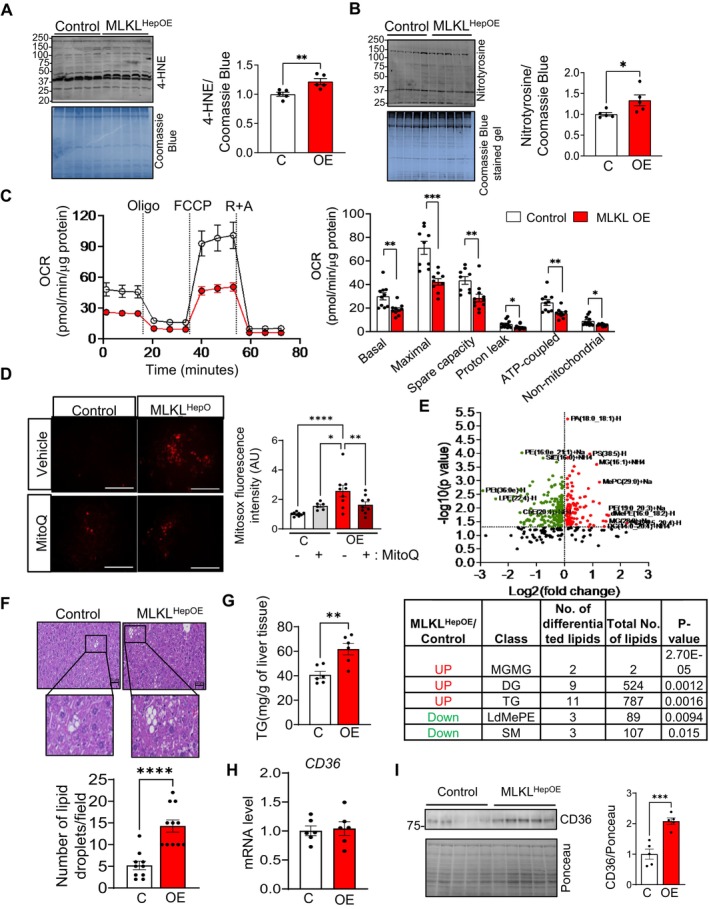
MLKL overexpression induces mitochondrial dysfunction and alters lipid metabolism in the liver. (A, B) Western blots (*left*) and quantification (*right*) of 4‐HNE (A) and nitrotyrosine (B) in control *(C)* and MLKL^HepOE^ *(OE)* livers; Coomassie Blue as loading control. (C) Mitochondrial respiration in control and MLKL‐overexpressing AML12 cells. *Left*: Representative OCR trace with oligomycin, FCCP, and rotenone/antimycin A. *Right*: Quantified respiratory parameters. (D) AML12 cells overexpressing MLKL treated with vehicle or MitoQ. *Left*: MitoSOX Red staining. *Right*: Fluorescence intensity (fold change). Scale bar: 100 μm. (E) Volcano plot of lipidomic changes in MLKL^HepOE^ vs. control livers; red, upregulated; green, downregulated; black, non‐significant. Table summarizes altered lipid classes. (F) Liver histology (13 months post‐injection). Top: H&E staining with enlarged insets showing lipid droplets. Bottom: Quantification of lipid droplets per field. (G) Hepatic triglyceride content. (H, I) CD36 expression by qPCR (H) and Western blot (I); quantification normalized to Ponceau stain. *n* = 4–6 mice/group; data shown as fold change (control, white; MLKL^HepOE^, red) and mean ± SEM. Statistical analysis by two‐tailed unpaired *t*‐test. ****p < 0.0001, ***p < 0.001, **p < 0.01, *p < 0.05.

Dysregulated autophagy, NF‐κB activation, and mitochondrial dysfunction are known inducers of senescence during aging (Miwa et al. 2022). To assess whether these pathways contribute to MLKL‐induced senescence, we first examined autophagy and NF‐κB activation markers in MLKL^HepOE^ livers. Both pathways were comparable between MLKL^HepOE^ and controls, suggesting they do not drive senescence (Figure S3A,B). In contrast, oxidative stress markers 4‐Hydroxynonenal (4‐HNE) and Nitrotyrosine (NT) were significantly elevated in MLKL^HepOE^ livers (Figure 4A,B). To assess mitochondrial function, a mitochondrial stress test in MLKL‐overexpressing AML12 cells revealed significant reductions in basal, maximal, and ATP‐linked respiration, spare respiratory capacity, proton leak, and non‐mitochondrial respiration (Figure 4C). MLKL overexpression increased mitochondrial reactive oxygen species (ROS) levels by 2.5‐fold, as measured by MitoSOX Red staining, whereas mitochondria‐targeted antioxidant Mitoquinol Mesylate (MitoQ) treatment reduced ROS levels to those comparable to untreated controls (Figure 4D). Notably, MitoQ treatment also improved mitochondrial respiration, as assessed by the mitochondrial stress test (Figure S3C), indicating that MLKL‐induced mitochondrial dysfunction is driven, at least in part, by oxidative stress.

Given the link between mitochondrial dysfunction, oxidative stress, and senescence, we performed targeted proteomics of mitochondrial proteins to define a potential involvement of mitochondrial dysfunction and oxidative stress in the induction of senescence in MLKL^HepOE^ mouse liver. Six of 25 Krebs cycle proteins and 10 of 31 β‐oxidation proteins were upregulated, indicating altered mitochondrial metabolism (Figure S3D), while other mitochondrial pathways remained unchanged (Figure S3E, Table S5). Lipidomics analysis revealed significant changes, with increased monoacylglycerophosphoglycerol (MGMG), diacylglycerol (DG), and triacylglycerol (TG), and decreased lysodimethylphosphatidylethanolamine (LdMePE) and sphingomyelin (SM) in MLKL^HepOE^ livers (Figure 4E, Table S6), altering disrupted lipid metabolism.

Cellular senescence in hepatocytes promotes steatosis, which contributes to MASLD pathology in aging (Ogrodnik et al. 2017). Histological analysis and triglyceride measurements at 5.5 months did not reveal significant differences in lipid accumulation or fibrosis markers between MLKL^HepOE^ and control mice (Figure S3F,G), indicating that short‐term MLKL overexpression does not cause steatosis, fibrosis, or liver injury. However, increased lipid droplets and triglyceride levels were observed at 13 months of age (Figure 4F,G). These findings suggest that MLKL overexpression promotes fat accumulation over time. We further checked expression of CD36, a major fatty acid transporter that is elevated in senescent cells (Chong et al. 2018). At 13 months of age, when hepatic steatosis is evident, CD36 transcript levels were comparable between groups; however, CD36 protein abundance increased approximately 2‐fold in MLKL^HepOE^ livers (Figure 4H,I). In contrast, at 5.5 months of age, when steatosis is not yet apparent, CD36 mRNA levels were elevated without a corresponding increase in protein expression (Figure S3H,I). Together, these findings suggest that MLKL overexpression promotes mitochondrial dysfunction, oxidative stress, and metabolic dysfunction.

### 3.6 MLKL Overexpression Alters Mitochondrial Dynamics in the Liver

**FIGURE 5:**
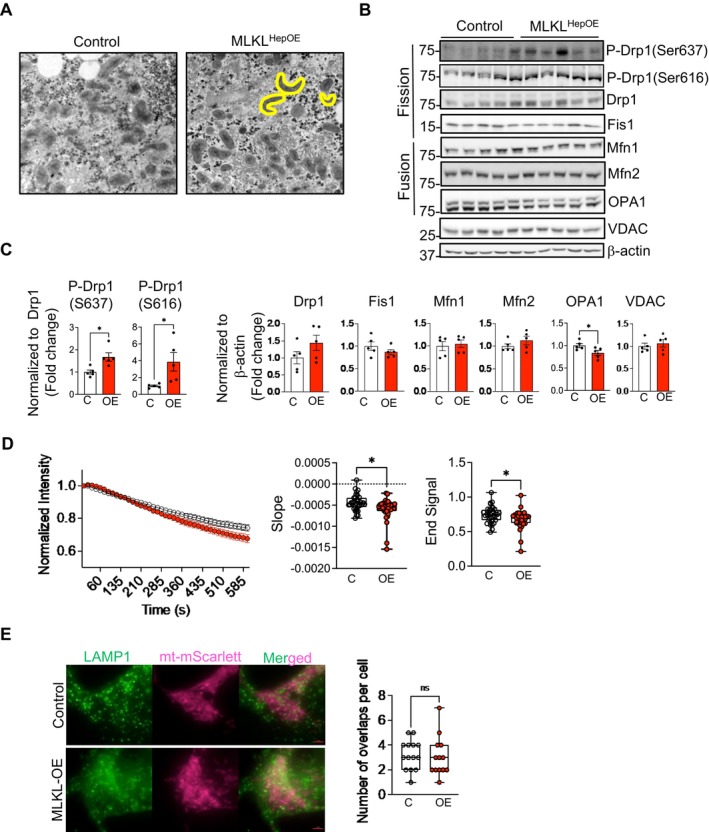
Hepatic MLKL overexpression alters mitochondrial morphology and dynamics. (A) Transmission electron microscopy of liver tissue from control *(C)* and MLKL^HepOE^ *(OE)* mice (*n* = 5/group) (1000×). (B) Western blot of mitochondrial fusion and fission proteins, with VDAC and β‐actin as controls. (C) Quantification of protein levels normalized to β‐actin. (D) *Left*: Photoactivatable GFP (paGFP) signal over 24 h in control and MLKL‐overexpressing AML12 cells. *Right*: Quantification (slope and end signal). (E) *Left*: Colocalization of LAMP1 (lysosome) with mt‐mScarlett (mitochondria, magenta). *Right*: Quantification of LAMP1‐mitochondria overlaps. Data are mean ± SEM; control (white) and MLKL^HepOE^ (red). Statistical analysis by two‐tailed unpaired *t*‐test. *p < 0.05; ns, not significant.

Transmission electron microscopy of liver tissues revealed altered mitochondrial morphology in MLKL^HepOE^ mice compared to control mice (Figure 5A). Although mitochondrial fragmentation or swelling was not observed, a subset showed abnormal C‐shaped morphology without changes in mitochondrial area (Figure S4A), suggesting altered mitochondrial dynamics. To further assess this, we examined key fusion proteins (mitofusin 1, Mfn1; Mfn2; optic atrophy 1, OPA1) and fission proteins [Fis1, Drp1, and phospho‐Drp1 at Ser637 (inhibitory) and Ser616 (pro‐fission)]. MLKL overexpression significantly increased the ratios of phospho‐Drp1 (Ser637 and Ser616) to total Drp1 (Figure 5B,C), while Mfn1, Mfn2, and Fis1 levels were unchanged. Notably, OPA1 levels were markedly reduced in MLKL^HepOE^ livers. VDAC levels remained similar between groups, indicating mitochondrial mass was unaffected. Together, these results suggest that MLKL overexpression disrupts mitochondrial dynamics.

To directly test MLKL's role in mitochondrial dynamics, we measured the spread of matrix‐targeted photoactivatable GFP (paGFP) signal 24 h after transfection in control and MLKL‐overexpressing AML12 cells. MLKL‐overexpressing cells exhibited a steeper decline in paGFP signal, suggesting enhanced mitochondrial trafficking or dynamics (Figure 5D). To assess whether altered dynamics impacted mitophagy, we measured colocalization of the endolysosomal marker LAMP1 with the mitochondrial marker mt‐mScarlet. Representative images showed regions of overlap (yellow) in both control and MLKL‐overexpressing cells (Figure 5E, *left*). Quantification revealed no significant difference in LAMP1/mt‐mScarlet interactions per cell (Figure 5E, *right*), indicating no change in mitophagy. Additionally, Parkin and PINK1 expression levels were similar in control and MLKL^HepOE^ livers (Figure S4B). These findings suggest that MLKL alters mitochondrial morphology independently of mitophagy.

### 3.7 MLKL Overexpression Enhances EV Release Without Inducing Cell Death

**FIGURE 6:**
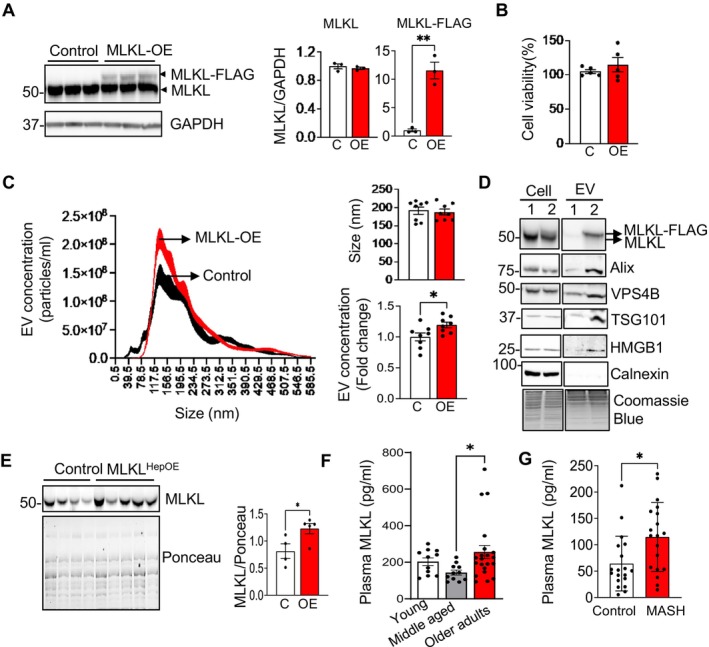
Hepatocyte MLKL overexpression promotes EV release and increases circulating MLKL. AML12 cells were transfected with empty vector (pcDNA, control, white) or pcDNA‐MLKL‐FLAG (MLKL‐OE, red) for 24 h. (A) *Left*: Western blot of MLKL (FLAG and endogenous) and GAPDH. Right: Quantification normalized to GAPDH. (B) Cell viability (CCK‐8 assay). (C) *Left*: Nanoparticle tracking analysis of EVs. *Right*: EV size and concentration. (D) Western blot of cell lysates and EVs for EV markers (ALIX, VPS4B, TSG101), HMGB1, MLKL, and calnexin (ER marker); Coomassie Blue as loading control. (E) Plasma MLKL in control and MLKL^HepOE^ mice (4 months post‐expression); blot and quantification (Ponceau control; *n* = 4–5/group). (F) Plasma MLKL levels in humans across age groups (young: 21–45, middle‐aged: 45–65, older: > 65; *n* = 11/group for young and middle‐aged, *n* = 21 for older) measured by ELISA. (G) Plasma MLKL in healthy controls (white) vs. MASH patients (red) (*n* = 20/group; ELISA). Data are mean ± SEM. Statistics: Two‐tailed unpaired *t*‐test (A–E, G) or one‐way ANOVA (F). **p < 0.01, *p < 0.05.

Given that MLKL overexpression did not induce necroptosis despite MLKL oligomer formation, we further investigated its impact in the AML12 mouse hepatocyte cell line. Studies suggest that MLKL‐dependent EV biogenesis, independent of necroptosis, is a potential mechanism that prevents cell death (Yoon et al. 2017). Transfection with pcDNA‐MLKL‐FLAG resulted in a 10‐fold increase in MLKL‐FLAG expression compared to control cells (Figure 6A). MLKL overexpression did not induce cell death (Figure 6B) but enhanced EV release, as measured by nanoparticle tracking analysis, without altering EV size (Figure 6C). MLKL overexpression caused continuous release of MLKL‐FLAG in EVs at 12, 24, and 36 h, associated with a decline in intracellular MLKL‐FLAG over time (Figure S5A). Conversely, knocking down MLKL in a human liver cancer cell line HepG2, which exhibits higher MLKL expression compared to the normal human liver cell line THLE2, resulted in a reduction in both the concentration and size of EVs, as determined by NTA (Figure S5B–D). Western blotting of EVs from MLKL‐overexpressing AML12 cells showed increased EV markers (ALIX, VPS4B, TSG101), exogenous MLKL‐FLAG, and the pro‐inflammatory DAMP HMGB1, compared to controls (Figure 6D). Consistent with these observations, plasma samples from MLKL^HepOE^ mice contained higher levels of circulating MLKL compared to control mice (Figure 6E). Importantly, plasma MLKL concentrations were significantly elevated in aged humans when compared to the younger counterparts (Figure 6F). Additionally, we observed that MLKL levels in the plasma of MASH patients are significantly higher than in healthy individuals (Figure 6G). Thus, while MLKL‐driven EV release could serve as a cell‐protective mechanism that limits hepatocyte death, proinflammatory cargo present in these EVs could possibly promote paracrine effects on surrounding or distal cells.

## 4 Discussion

Aging is characterized by chronic low‐grade inflammation (inflammaging) (Franceschi and Campisi 2014) and metabolic dysfunction, which together promote the development of liver diseases such as MASLD and HCC. Although MLKL is best known as the terminal executioner of RIPK3‐dependent necroptosis, accumulating evidence supports important non‐necroptotic functions of MLK, including endosomal trafficking, EV biogenesis, and autophagy (Wu et al. 2020; Yoon et al. 2017). In this study, we identify a previously unrecognized role of hepatocyte MLKL as a contributing driver of mitochondrial dysfunction, oxidative stress, cellular senescence, and paracrine inflammatory signaling that together drive liver inflammaging (graphical abstract).

Using hepatocyte‐specific MLKL overexpression in young mice as a gain‐of‐function model, we demonstrate that elevated hepatocyte MLKL is sufficient to induce hallmarks of liver aging independent of necroptotic cell death. MLKL protein levels increase ~2‐fold in hepatocytes during physiological aging, and the level achieved in MLKL^HepOE^ mice (~3.5‐fold) falls within the range of the physiological increase observed in aged hepatocytes. Importantly, our longitudinal data show that hepatic MLKL does not increase gradually across the lifespan but instead rises in a compressed late‐life window beginning around 18 months (Mohammed, Thadathil, et al. 2021), a pattern more consistent with our model than a gradual lifelong increase. The MLKL^HepOE^ model was designed as a mechanistic sufficiency approach, not to fully recapitulate physiological aging, but to define MLKL‐dependent mechanisms that contribute to liver inflammaging. MLKL was maintained at elevated levels for four months in young mice to isolate hepatocyte‐specific mechanisms while minimizing confounding systemic effects of advanced age. MLKL overexpression recapitulates multiple features of hepatic aging, including altered mitochondrial dysfunction and dynamics, increased oxidative stress, induction of p16 and p21 expression, and robust expression of SASP factors. MLKL^HepOE^ mice exhibited elevated circulating MLKL consistent with increased serum MLKL observed in aged humans, further linking MLKL to the systemic aging phenotype. Although hepatocyte MLKL elevation is unlikely to represent the sole driver of aging, our data position MLKL as an amplifying node within the aging liver, and the MLKL^HepOE^ mouse provides a controlled system to define MLKL‐dependent hepatocyte mechanisms independent of the complex systemic milieu of aged animals.

Despite the extensive literature implicating necroptosis pathway activation in MASLD and aging (Afonso et al. 2015; Gautheron et al. 2015; Mohammed, Thadathil, et al. 2021), hepatocyte MLKL overexpression did not induce canonical necroptosis, consistent with literature suggesting that liver necroptosis primarily arises from non‐parenchymal cells (Dara et al. 2016), rather than hepatocytes. MLKL^HepOE^ livers lacked RIPK3 expression, showed no detectable phosphorylation of RIPK3 or MLKL, and did not show liver injury or elevated liver enzymes. These findings are consistent with reports showing epigenetic silencing of RIPK3 in hepatocytes (Preston et al. 2022). Interestingly, MLKL oligomerization increased in MLKL^HepOE^ livers despite the absence of RIPK3, suggesting alternative regulatory mechanisms (e.g., TAM kinase phosphorylation, Trx1‐related redox control, or intrinsic activation of MLKL N‐terminus) that permit oligomer formation without necroptotic execution (Dondelinger et al. 2014; Najafov et al. 2019; Reynoso et al. 2017). Because oligomerization alone is insufficient to trigger cell death in the absence of membrane translocation and pore formation (Petrie et al. 2017); MLKL can adopt non‐lethal signaling roles in hepatocytes.

Mechanistically, our data supports a model in which hepatocyte MLKL drives mitochondrial dysfunction as an initiating event. MLKL overexpression impaired mitochondrial respiration, disrupted mitochondrial dynamics, altered mitochondrial morphology, and increased mitochondrial reactive oxygen species (ROS). Specifically, MLKL^HepOE^ livers increased Drp1 phosphorylation and reduced OPA1 expression, indicating excessive mitochondrial fission relative to fusion. This imbalance was associated with the accumulation of structurally abnormal C‐shaped mitochondria, a morphology linked to pathological fission and occurred without compensatory activation of mitophagy, suggesting persistence of dysfunctional mitochondria (Mahajan et al. 2021), Importantly, antioxidant rescue with MitoQ attenuated mitochondrial oxidative stress and improved mitochondrial respiration, supporting mitochondrial ROS as a downstream consequence of MLKL overexpression. These findings align with the prior work demonstrating mitochondrial localization of MLKL (Y. Wang et al. 2025), and its deletion in hepatocytes resulted in improved respiration, β‐oxidation, and reduced oxidative DNA damage (Majdi et al. 2020; Xu et al. 2023). Additionally, these results align with previous work demonstrating that TPP conjugates, such as MitoQ, CoQ, Resveratrol, and metformin, impact mitochondrial respiration based on the initial membrane potential (Zielonka et al. 2017). Highly functional mitochondria can be negatively impacted, while lower performing mitochondria see dramatic improvement (Bubak et al. 2024; Gottwald et al. 2018). It remains unclear if this is a direct effect on mitochondrial function, a result of accelerated mitochondrial turnover, or a combination of both.

Mitochondrial dysfunction and oxidative stress are well established drivers of cellular senescence (Miwa et al. 2022). Consistent with this paradigm, MLKL‐driven mitochondrial defects were associated with increased p16, p21, induction of pro‐inflammatory SASP factors, and steatosis, mirroring transcriptional and phenotypic changes observed in aging liver (Mohammed et al. 2025; Mohammed, Thadathil, et al. 2021; Ogrodnik et al. 2017). Together, these findings provide converging evidence linking hepatocyte MLKL upregulation to hepatic senescence, with mitochondrial oxidative stress as a contributing mechanism, independent of necroptosis. Definitive causal testing of the mitochondria‐to‐senescence axis in vivo will require hepatocyte‐targeted interventions beyond the scope of the present study.

A key finding of this study is that hepatocyte‐specific MLKL‐driven senescence is not restricted to hepatocytes but extends to multiple liver cell types including macrophages, stellate cells, and endothelial cells. While hepatocytes contribute substantially to the total senescent burden due to their abundance, macrophages exhibited the highest proportion of senescence markers p16 and p21, identifying them as a senescence enriched population. Whether hepatocytes are the primary cells that undergo senescence first in response to mitochondrial oxidative stress, which is subsequently propagated to NPCs, or whether both hepatocytes and NPCs undergo senescence simultaneously remains unclear. Based on our observation that MLKL overexpression in hepatocytes does not result in sustained intracellular accumulation, likely due to EV‐mediated export, we propose a non‐cell‐autonomous model of senescence. In this model, MLKL‐induced mitochondrial dysfunction and oxidative stress in hepatocytes act as initiating signals that promote senescence in both hepatocytes and NPCs through bystander effects (graphical abstract).

Mechanistically, mitochondrial oxidative stress is known to promote HMGB1 extracellular release, and scavenging mitochondrial ROS using MitoQ reduces HMGB1 release in hepatic ischemia–reperfusion injury (van Golen et al. 2019), supporting a causal link between mitochondrial stress and HMGB1 secretion. Consistent with this framework, we observed that hepatocyte MLKL overexpression increased EV release enriched in HMGB1, a known inducer of senescence (Davalos et al. 2013). While HMGB1 is one candidate identified in our study, other secreted factors, including TGF‐β, IL‐1, and IL‐6, are also known to induce senescence in neighboring cells through paracrine bystander effects (Hubackova et al. 2012). Together, these findings support a model in which MLKL‐driven mitochondrial stress in hepatocytes promotes HMGB1 and cytokine release, a mechanism that remains to be directly tested for its role in driving multicellular senescence and chronic inflammation in the liver.

Proteomic pathway analysis revealed enrichment of signaling networks associated with HCC, hepatic lipidosis, and unexpectedly neurodegenerative diseases such as Amyotrophic Lateral Sclerosis (ALS) and Huntington's disease. Given the critical role of inflammation in HCC initiation and progression (Yang et al. 2019), the enrichment of oncogenic pathways supports a tumor‐promoting role for MLKL‐driven inflammaging. Consistent with this, both global and hepatocyte‐specific deletion of MLKL reduced HCC incidence in diet‐induced MASH models (Mohammed et al. 2023; Ohene‐Marfo et al. 2025). Enrichment of lipidosis‐related pathways aligns with the observed steatosis and altered lipid metabolism, indicating metabolic dysfunction independent of cell death. Notably, the enrichment of ALS‐ and Huntington's disease‐related pathways raises the possibility that hepatic MLKL overexpression exerts systemic effects, potentially via the release of inflammatory mediators or EVs influencing distant organs such as the brain. This aligns with emerging evidence linking liver pathology to neuroinflammation, cognitive decline, and Alzheimer's disease (Chen et al. 2026), supporting a role for liver‐brain crosstalk in aging and disease. Together, these findings suggest that MLKL‐mediated signaling in hepatocytes intersects with oncogenic, metabolic, and neurodegenerative pathways, highlighting the broad pathological impact of MLKL dysregulation.

In summary, our study identifies a non‐necroptotic function of hepatocyte MLKL as a driver of liver inflammaging. We propose a model in which increased hepatocyte MLKL initiates mitochondrial dysfunction, oxidative stress, and propagates multicellular senescence through paracrine signaling, particularly affecting macrophages. These findings establish MLKL as a regulator of mitochondrial and inflammatory homeostasis independent of its canonical role in necroptosis and highlight MLKL‐driven intercellular communication as a potential therapeutic target in age‐associated liver disease.

### 4.1 Limitations of the Study

First, although hepatocyte‐specific MLKL overexpression induced robust senescence‐associated phenotypes, the MLKL^HepOE^ model was designed as a mechanistic sufficiency tool and is not intended to fully recapitulate the dynamics of physiological MLKL elevation during aging. Future studies incorporating aged reference groups, complementary loss‐of‐function models, and dose‐controlled approaches will be required to fully validate the physiological relevance of these findings. Second, while our data support a model of hepatocyte‐driven, multicellular senescence, the direct causal role of hepatocyte‐derived EVs and inflammatory signals in inducing secondary senescence in non‐parenchymal cells was not definitively established in vivo. Third, while in vitro MitoQ rescue experiments demonstrate that mitochondrial ROS contribute to MLKL‐induced bioenergetic defects, the causal link between mitochondrial dysfunction and senescence in vivo remains to be directly established. Hepatocyte‐targeted in vivo rescue approaches will be required to fully resolve this causal chain. Fourth, although RNAscope analysis revealed cell type‐specific differences in senescence marker expression, the functional contribution of individual cell populations, particularly macrophages as a senescence‐enriched compartment, to liver inflammation and pathology remains to be directly tested.

## Author Contributions

S.M., C.J., T.P., S.B., P.O.‐M. and K.F.K. performed experiments, analyzed data, prepared figures, and contributed to manuscript writing and editing. C.G. and J.D.W. analyzed lipidomics data. K.P. performed ELISA for MLKL in human MASH samples provided by C.H.; Z.P., A.S., Z.Y., and N.A. performed and analyzed label‐free quantitative proteomics of liver samples. A.T. assisted with animal studies. M.K. conducted and analyzed targeted mitochondrial proteomics. T.L. assisted with AAV8 viral injections. B.H. provided input on EV isolation and characterization protocols. A.Y. provided the young and aged human plasma samples. V.G. provided suggestions for senescence marker analysis. T.L.L. designed mitochondrial reporter assays. S.S.D. designed the experiments, supervised the research, and wrote the manuscript. All authors reviewed and edited the manuscript.

## Funding

This work was supported by the National Institutes of Health (Grants R01AG059718 and R03CA262044), Harold Hamm Diabetes Center–Stephenson Cancer Center Team Science, Oklahoma Center for Adult Stem Cell Research (Grant 251004), Oklahoma Center for the Advancement of Science and Technology (Grant HF21‐009) to S.S.D., National Institute of General Medical Sciences (Grants R35GM137921 to T.L.L., RF1AG068283 to V.G., R01AG075834, and R21AG080775 to A.Y.), American Heart Association (Grant 966924), and Reynolds Foundation to A.Y. Salary support for S.M. was provided by Oklahoma Center for the Advancement of Science and Technology (OCAST) Health Research Postdoctoral Fellowship. N.A. gratefully acknowledges initial funding from the Office of the Vice President for Research and Partnerships at the University of Oklahoma for establishing the Proteomics Core Facility.

## Conflicts of Interest

The authors declare no conflicts of interest.

## Supporting information


**Figure S1:** (A) Schematic representation of the *Mlkl* genomic locus and the strategy used to generate MLKL^HepOE^ mice. Exons are depicted as boxes, with loxP sites flanking exon 2 shown as blue triangles. To enable conditional overexpression, a donor DNA construct encoding a stop codon followed by the *Mlkl*‐3xFlag sequence was inserted into the *Rosa26* locus via CRISPR‐Cas9–mediated homologous recombination. (B) Western blot analysis of liver tissue lysates from control and MLKL^HepOE^ mice showing MLKL overexpression, 2 weeks post virus injection. β‐actin serves as a loading control. (C) Body weight comparison between control and MLKL^HepOE^ mice. (D) Absolute liver weight (g) and liver‐to‐body weight ratio (% of body mass) in control and MLKL^HepOE^ mice (E) Weights of epididymal white adipose tissue (eWAT), heart, kidney, lungs, gastrocnemius muscle (Gastroc), and spleen, expressed as a percentage of body weight (% BW), in control and MLKL^HepOE^ mice. (F) qPCR analysis of *Mlkl* mRNA levels in the livers of control and MLKL^HepOE^ mice. (G) qPCR analysis of *albumin* and *F4/80* levels in isolated hepatocytes (Hep) and non‐parenchymal cells (NPCs) isolated from control and MLKL^HepOE^ mice. (H) *Top*: Western blot analysis showing MLKL oligomers in liver tissue lysates from control and MLK^HepOE^ mice. *Bottom*: Quantification of MLKL oligomers normalized to β‐tubulin. (I) RAW264.7 cells were polarized to M1 phenotype by LPS treatment, and treated with vehicle, RIPK1 inhibitor necrostatin 1 s (Nec‐1 s, 30 μM) or RIPK3 inhibitor GSK872 (1 μM) for 30 min prior to the addition of TBZ for 24 h. Protein expression of p‐MLKL and MLKL were checked by western blotting, β‐tubulin is used as loading control. (J) *Left*: Representative images of TUNEL staining (green) with DAPI counterstaining (blue) in liver sections from control, MLKL^HepOE^ mice, and a positive control (DNase treated liver sections). Arrows indicate TUNEL‐positive nuclei. *Right*: Quantification of TUNEL‐positive cells per field (4 fields/section; *n* = 3 animals per group). (K) Western blot analysis of cleaved PARP (Cl. PARP), cleaved caspase 3 (Cl. Caspase 3), and total caspase 3 protein levels in liver lysates of control and MLKL^HepOE^ mice. β‐tubulin serves as loading control. Control (white) and MLKL^HepOE^ (red). Data are presented as mean ± SEM from *n* = 5–9 mice/group except J. Statistical significance was determined by two‐tailed unpaired *t*‐test for C‐F, H or One‐way ANOVA for G. ****p < 0.0001, ***p < 0.001, *p < 0.05, ns: not significant.
**Figure S2:** Data from control and MLKL^HepOE^ mice livers. qPCR analysis of (A) SASP factors. (B) M1 macrophage markers (C) M2 macrophage markers. (D) Cell type abundance (absolute) ‐Total number of a certain cell type within each group shown to represent the cell type abundance. Absolute p16/p21 double‐positive counts (E) p16 or (F) p21 positive cell numbers within a certain cell type is shown. Total (G) p16 or (H) p21 copy numbers in a certain cell type. (I) p16 or (J) p21 copy numbers within 100 cells in each cell type. For (A–C) Control (C, white) and MLKL^HepOE^ (OE, red). For (C–H) Red bars represent macrophages, white bars represent endothelial cells, pink bars represent hepatic stellate cells and green bars represent hepatocytes. Data are presented as mean ± SEM from *n* = 7–9 mice/group (A–C); *n* = 4 mice/group (D‐H). Statistical significance was determined by two‐tailed unpaired *t*‐test. **p < 0.01, *p < 0.05, ns: not significant.
**Figure S3:** Western blot analysis of liver tissue lysates from control (C, white) and *MLKL* ^*HepOE*^ (OE, red) mice showing (A) *Left*: LC3‐I, LC3‐II and β‐actin (loading control), *Right*: Ratio of LC3‐II/LC3‐I represented as fold change, (B) *Left*: Phosphorylated p65 (Phospho‐p65), total p65, *Right*: Phospho‐p65/p65 ratio, presented as fold change. (C) AML12 cells were transfected with empty vector (pcDNA) or MLKL‐Flag and treated with vehicle [EtOH: H_2_O (1:1)] or MitoQ (250 nM). MitoQ treatment was initiated 6 h post‐transfection and maintained for 24 h prior to analysis. Mitochondrial respiration was assessed using the Seahorse Mito Stress Test. (D) Heat maps of proteins involved in Kreb's and TCA cycle obtained by targeted mitochondrial proteomics of liver tissues from MLKLHepOE vs. control mice. Red represents up‐regulation and white denotes no significant change. Asterisks (*) denote statistically significant changes between the two groups. (E) Heatmap of differentially expressed proteins in the mitochondrial pathways in the livers of *MLKL* ^*HepOE*^ mice compared to control mice, as determined by targeted proteomics. (F) Representative images of H&E‐stained liver sections from short term (5.5 months) MLKL^HepOE^ mice. Scale bar = 50 μm. Magnification: 100×. The graph on the bottom shows quantification of lipid droplets per field (*n* = 3–4 fields per animal, 3 animals per group) (G) Triglyceride (TG) content (mg/g) of liver tissue (H‐I) qPCR (H) and western blot analysis (I) of CD36 in livers from 5.5‐month‐old control and MLKL^HepOE^ mice. *Right*: Quantification of CD36 normalized to Ponceau staining. Data from control (C, white) and MLKL^HepOE^ (OE, red) mice (*n* = 5–7/group). All data are presented as mean ± SEM from *n* = 5 mice/group. Statistical significance was determined by two‐tailed unpaired *t*‐test, except C (Two‐way ANOVA). ****p < 0.0001, ***p < 0.001, **p < 0.01, *p < 0.05, ns: not significant.
**Figure S4:** (A) Mitochondrial area (μm^2^) in liver sections of control (black circles) and MLKL^HepOE^ (red dots) mice. Each dot represents an individual mitochondrion. (B) Western blot analysis showing protein levels of PARKIN, full‐length PINK1, and cleaved PINK1 in liver lysates from control and MLKL^HepOE^ mice. β‐actin serves as a loading control. All data are presented as mean ± SEM from *n* = 5 mice/group. Statistical significance was determined by two‐tailed unpaired *t*‐test. ns: not significant.
**Figure S5:** (A) AML12 cells were transfected with either empty vector (pcDNA, control) or pcDNA‐MLKL‐FLAG (MLKL‐OE) for 12, 24 or 36 h. The cell culture supernatant was used for isolating EVs. Lysates were prepared from the cells and EVs, and western blot analysis was performed for EV marker (Alix), ER marker (Calnexin) and MLKL. Coomassie blue stained gel is used as loading control. (B) Western blot analysis of basal MLKL protein expression in THLE2 (normal) and HepG2 (liver cancer) cell lines. β‐tubulin is used as a loading control. (C) *Left*: Western blot analysis of MLKL protein expression in HepG2 cells transfected with either siControl or siMLKL. β‐tubulin serves as loading control. *Right*: The bar graph shows quantification of MLKL protein levels normalized to β‐tubulin, presented as fold change (*n* = 3 independent experiments). (D) Nanoparticle Tracking Analysis (NTA) of EVs isolated from HepG2 cells transfected with siControl (pink) or siMLKL (green). The left panel shows the EV size distribution, the middle panel presents total EV concentration (particles/ml), and the right panel displays average EV diameter (nm) (*n* = 3 independent experiments). Statistical significance was determined by two‐tailed unpaired *t*‐test. ***p < 0.001, *p < 0.05.

**Table S1:** List of primer sequences used for quantitative RT PCR analysis.

**Table S2:** Details of patient samples used for human plasma MLKL analysis in MASH patients.

**Table S3:** Details of samples used for human plasma MLKL analysis in young and old cohort.

**Table S4:** List of significantly altered proteins in untargeted label free quantitative proteomic analysis.

**Table S5:** List of proteins identified by targeted mitochondrial proteomics.

**Table S6:** List of differentially regulated lipid species in lipidomic analysis.

## Data Availability

Proteomics data can be found in the MassIVE database via MSV000097694. Additional data supporting the study's findings are provided within the manuscript and its Supporting Information.
