# Correction to “A Non‐Canonical Role for Hepatocyte MLKL in Promoting Mitochondrial Dysfunction and Senescence in the Aging Liver”

**DOI:** 10.1111/acel.70746

**Published:** 2026-10-02

Mohammed, S. , C. Jiang , T. Pennington , et al. 2026. “A Non‐Canonical Role for Hepatocyte MLKL in Promoting Mitochondrial Dysfunction and Senescence in the Aging Liver.” Aging Cell 25, no. 7: e70618. 10.1111/acel.70618.PMC1333175142399678

The following funding information for one of the co‐authors was inadvertently missed from the original published manuscript. “V.G. is a recipient of a Research Career Scientist award IK6 BX006026.” The updated Funding section is as follows:

This work was supported by the National Institutes of Health (Grants R01AG059718 and R03CA262044), Harold Hamm Diabetes Center–Stephenson Cancer Center Team Science, Oklahoma Center for Adult Stem Cell Research (Grant 251004), Oklahoma Center for the Advancement of Science and Technology (Grant HF21‐009) to S.S.D., National Institute of General Medical Sciences (Grants R35GM137921 to T.L.L., RF1AG068283 to V.G., R01AG075834, and R21AG080775 to A.Y.), American Heart Association (Grant 966924), and Reynolds Foundation to A.Y. V.G. is a recipient of a Research Career Scientist award IK6 BX006026. Salary support for S.M. was provided by Oklahoma Center for the Advancement of Science and Technology (OCAST) Health Research Postdoctoral Fellowship. N.A. gratefully acknowledges initial funding from the Office of the Vice President for Research and Partnerships at the University of Oklahoma for establishing the Proteomics Core Facility.

We apologize for this error.

