# Supplementary material for: A Non‐Canonical Role for Hepatocyte MLKL in Promoting Mitochondrial Dysfunction and Senescence in the Aging Liver
Source: Aging Cell. 2026 Jul 3;25(7):e70618. doi: 10.1111/acel.70618 (PMC13331751; doi:10.1111/acel.70618)
Supplement: Supplementary file 1 — Figure S1: (A) Schematic representation of the Mlkl genomic locus and the strategy used to generate MLKLHepOE mice. Exons are depicted as boxes, with loxP sites flanking exon 2 shown as blue triangles. To enable conditional overexpression, a donor DNA construct encoding a stop codon followed by the Mlkl‐3xFlag sequence was inserted into the Rosa26 locus via CRISPR‐Cas9–mediated homologous recombination. (B) Western blot analysis of liver tissue lysates from control and MLKLHepOE mice showing MLKL overexpression, 2 weeks post virus injection. β‐actin serves as a loading control. (C) Body weight comparison between control and MLKLHepOE mice. (D) Absolute liver weight (g) and liver‐to‐body weight ratio (% of body mass) in control and MLKLHepOE mice (E) Weights of epididymal white adipose tissue (eWAT), heart, kidney, lungs, gastrocnemius muscle (Gastroc), and spleen, expressed as a percentage of body weight (% BW), in control and MLKLHepOE mice. (F) qPCR analysis of Mlkl mRNA levels in the livers of control and MLKLHepOE mice. (G) qPCR analysis of albumin and F4/80 levels in isolated hepatocytes (Hep) and non‐parenchymal cells (NPCs) isolated from control and MLKLHepOE mice. (H) Top: Western blot analysis showing MLKL oligomers in liver tissue lysates from control and MLKHepOE mice. Bottom: Quantification of MLKL oligomers normalized to β‐tubulin. (I) RAW264.7 cells were polarized to M1 phenotype by LPS treatment, and treated with vehicle, RIPK1 inhibitor necrostatin 1 s (Nec‐1 s, 30 μM) or RIPK3 inhibitor GSK872 (1 μM) for 30 min prior to the addition of TBZ for 24 h. Protein expression of p‐MLKL and MLKL were checked by western blotting, β‐tubulin is used as loading control. (J) Left: Representative images of TUNEL staining (green) with DAPI counterstaining (blue) in liver sections from control, MLKLHepOE mice, and a positive control (DNase treated liver sections). Arrows indicate TUNEL‐positive nuclei. Right: Quantification of TUNEL‐positive cells per field (4 field [file ACEL-25-e70618-s001.docx]

**Supplementary Methods**

Western Blotting

In brief, 20 mg liver tissue were homogenized in lysis buffer [50 mM 4-(2-hydroxyethyl)-1-piperazineethanesulfonic acid (HEPES), pH 7.6; 150 mM sodium chloride; 20 mM sodium pyrophosphate; 20 mM β-glycerophosphate; 2 mM EDTA; 1% Nonidet P-40; 10% glycerol; 2 mM phenylmethylsulfonyl fluoride; and protease inhibitor cocktail (GoldBiotechnology, St Louis, MO)]. After centrifugation at 14000 RPM for 20 min, protein concentrations in the supernatant were determined using Bio-Rad protein assay dye reagent (Bio-Rad, Hercules, CA, USA). Western blotting was performed using 30 μg protein/well. Gels after western blotting were stained with Blazin' Blue™ Protein Gel Stain (#P-810-1, Gold Biotechnology), and the blots were stained with Ponceau S solution (#P7170-1L, Sigma-Aldrich) as per manufacturer’s instruction. Images were taken with the ChemiDoc imager (Bio-Rad) and quantified using ImageJ software (U.S. National Institutes of Health).The following primary antibodies were used: Flag (#F1804, Millipore), MLKL (#MABC604, Millipore), phospho-MLKL (#ab196436, Abcam), phospho-RIPK3 (S232 + T231) (#ab205421, Abcam), RIPK3 (#17563-1-AP, Proteintech), RIPK1 (#NBP1-77077,Novus Biologicals), Alix (#12422-1-AP, Proteintech), VPS4B (#17673-1-AP, Proteintech), TSG101 (#28283-1-AP, Proteintech), HMGB1 (#ab18256, Abcam), Calnexin (#10427-2-AP, Proteintech), LC3 (#14600-1-AP, Proteintech), Nitrotyrosine (#A-21285, Invitrogen), phospho-Drp1(Ser637) (#4867, Cell Signaling), phospho-Drp1(Ser616) (#PA5-64821, Thermo Scientific), Drp1(#12957-1-AP, Proteintech), Fis1 (#10956-1-AP, Proteintech), Mfn1 (#13798-1-AP, Proteintech), Mfn2 (#12186-1-AP, Proteintech), OPA1 (#ab42364, Abcam), VDAC (#ab15895, Abcam), Cleaved PARP (#13371-1-AP, Proteintech), Cleaved Caspase 3 (#9661, Cell Signaling Technology), Caspase 3 (#19677-1-AP, Proteintech), , phospho-p65 (#3033T, Cell Signaling Technology), p65 (#8242S, Cell Signaling Technology), p53 (#ab26, Abcam), PINK1 (#23274-1-AP, Proteintech), PARKIN (#14060-1-AP, Proteintech), p16 (#sc-74401, SantaCruz Biotechnology, TX, USA), p21 (#ab188224, Abcam), CD36 (#AF2519, R&D systems, MN, USA), β-actin (#A5441, Sigma-Aldrich), β-tubulin (#T5201, Sigma-Aldrich), GAPDH (#G8795, Sigma-Aldrich). HRP-linked anti-rabbit IgG, HRP-linked anti-mouse IgG and HRP-linked anti-rat IgG secondary antibodies were from Cell Signaling Technology.

Detection of 4-Hydroxynonenal (4-HNE) Adducts

For the detection of 4-HNE modified proteins, equal amounts of protein (40 μg/lane) were separated by SDS–PAGE, transferred to polyvinylidene difluoride membranes and treated with 250 mM sodium borohydride in 100 mM (3-(N-morpholino) propanesulfonic acid, MOPS), pH 8.0 for 15 min. The membrane was washed with water, followed by Tris buffered saline with Tween 20 (TBS-T), and blocked with 5% non-fat milk/TBS-T. The membrane was incubated with a 1:2000 dilution of polyclonal antibody against 4-HNE (gift from Dr. Luke Szweda, Oklahoma Medical Research Foundation). The antibody recognizes cysteine, lysine, and histidine 4-HNE protein adducts and is highly specific to 4-HNE derived protein adducts. This was followed by incubation with anti-rabbit IgG HRP conjugated antibody, and the blot was developed using ECL Western Blotting Substrate (ThermoFisher Scientific, Waltham, MA). Images were taken using a ChemiDoc imager (Bio-Rad, CA, USA) and quantified using ImageJ software (U.S. National Institutes of Health, Bethesda, MD, USA).

Immunofluorescence (IF) Staining

Paraffin sections were deparaffinized and rehydrated through xylene, graded ethanol (100%, 95%, 70%, 50%), and deionized water. Heat-induced antigen retrieval was performed, followed by permeabilization with 0.1% Tween-20 in PBS for 10 min. After PBS washing, sections were blocked with 2% BSA at room temperature for 60 min. Primary antibody F4/80 (NB600-404, Novus Biologicals, CO, USA) was diluted in 1% BSA (1: 400) and incubated at 4°C overnight. After PBS washing, Donkey anti-rat Alexa Fluor 488 (Abcam) secondary antibody was applied for 60 min at room temperature. The sections were washed with PBS and mounted with ProLong™ Diamond antifade mountant with DAPI (#P36962, Thermofisher scientific). Images were taken with Nikon TE2000-E microscope at 200 magnification, 3 random non-overlapping fields per sample were recorded. The percentage area of positive signal (green) per field was quantified using ImageJ software and was normalized to DAPI. The data are presented as fold change after normalization to control mice.

Quantitative Real-Time PCR (qPCR)

Twenty mg frozen liver tissue was used to isolate RNA using RNeasy Mini Kit (74106, Qiagen) as per manufacturer’s instruction. First-strand cDNA was synthesized, and the real-time-PCR was performed. The primers used are listed in Table S1. The calculations were conducted by a comparative method (2^−ΔΔCt^). β-microglobulin, β-actin, or hypoxanthine phosphoribosyltransferase 1 (HPRT) was used as controls.

Label Free Quantitative Proteomic Analysis of Liver

A total of 100 µg of liver lysate (n=5/group) was digested in-solution using Trypsin/LysC (#V5071, Promega, WI, USA) following the manufacturer’s instructions. Following digestion, the peptides were desalted with C18 Sep-Pak Plus cartridges (Waters, MA, USA). 100 µL of 0.1% formic acid was used to reconstitute the dried tryptic peptides to a final concentration of 1 µg/µL. Resuspended tryptic peptides (2 μL) were loaded onto a C18 trap column (150 μm × 3 cm, 3 μm resin, Acclaim™ PepMap™ 100 C18 HPLC Column, Thermo Scientific™, USA) using mobile phase A (0.1% formic acid in LC-MS grade water). The flow rate was 3 μl/min for 10 min, and separate peptides on an EASY-Spray™ HPLC analytical column (3 μm x 75 μm × 15 cm, Catalog # ES900 Thermo Scientific™, USA) at the rate of 350 nL/min. The total LC-MS/MS run time was 1 hour. The LC-MS/MS analysis was performed with a Dionex UltiMate® 3000 UHPLC system (Thermo Fisher Scientific, CA, USA) coupled to a Q Exactive HF-X mass spectrometer (Thermo Fisher Scientific, Waltham, MA).

The RAW MS files were searched against the UniProt reviewed mouse (Taxon ID: 10090) protein database, the Sequest algorithm within Proteome Discoverer v 2.4 (Thermo Fisher Scientific, San Jose, CA). Parameters used are listed as follows: trypsin enzyme cleavage specificity, 2 possible missed cleavages, 10 ppm mass tolerance for precursor ions, and 0.02 Da mass tolerance for fragment ions. The above search parameters permit dynamic modification of methionine oxidation (+15.9949 Da) and static modification of carbamidomethylation (+57.0215 Da) on cysteine. After the database search, peptide assignments were filtered down to a 1% FDR (false discovery rate). Label-free quantitation was performed using the Minora algorithm and the adjoining bioinformatics tools available in Proteome Discoverer. Statistically significant is defined as a 1.5-fold increase or decrease in abundance with a p-value <0.05.

Targeted Mitochondrial Proteomics

20 μg of total liver homogenate was separated ~1.5 cm into a 12.5% SDS-PAGE gel (Criterion, Bio-Rad, Berkeley, CA, USA). Gels were fixed and stained using GelCode Blue stain (Pierce, Appleton, WI, USA), and the entire lane was excised into ~1 mm³ sections. Gel pieces were sequentially washed, reduced with dithiothreitol (DTT), alkylated with iodoacetamide, and digested with trypsin. The resulting peptides were extracted using 50% methanol/10% formic acid, dried, reconstituted in 1% acetic acid, and analyzed via selected reaction monitoring (SRM) using a triple quadrupole mass spectrometer (TSQ Quantiva, Thermo Scientific) coupled to a splitless capillary HPLC system (Ultimate 3000, Thermo Scientific). Peptide quantification was performed using Skyline software, which aligned collision-induced dissociation transitions and quantified chromatographic peak areas. Protein abundance was calculated as the sum of all monitored peptide responses. Relative protein levels were normalized to a BSA internal standard and verified using normalization to housekeeping proteins.

Untargeted Lipidomics Analysis

Approximately 50 mg of liver tissue was homogenized in 1.5 mL chloroform:methanol (2:1, v/v) and 0.5 mL ultrapure water. Samples were ground, vortexed, and sonicated at 4 °C for 30 minutes, followed by centrifugation at 3,000 rpm for 10 minutes. The lower phase was collected, dried under nitrogen, and reconstituted in 200 μL isopropanol:methanol (1:1, v/v) containing LPC (12:0) as an internal standard. After centrifugation at 12,000 rpm for 10 minutes at 4 °C, supernatants were used for UPLC-MS analysis. QC samples were prepared by pooling equal volumes from each sample. Lipid separation was conducted on an ACQUITY UPLC BEH C18 column using a gradient of solvent A (60% acetonitrile, 40% water, 10 mM ammonium formate) and solvent B (90% isopropanol, 10% acetonitrile, 10 mM ammonium formate) at 0.3 mL/min. Analysis was performed using a Q Exactive mass spectrometer (Thermo Scientific) in both ESI+ and ESI– modes with standard parameters. Raw data were processed with LipidSearch for peak alignment and identification. Multivariate analysis was performed using SIMCA-P (v14.1), including PCA, PLS-DA, and OPLS-DA. Significantly altered lipids were identified using VIP > 1.5, fold change > 2, and p < 0.05.

Measurement of Mitochondrial Respiration

To measure mitochondrial respiration, AML12 cells were seeded at a density of 10,000 cells/well kept at 37 °C incubator with 5% CO_2_ for 24 h. The cells were transfected with either pcDNA or pcDNA-MLKL-Flag as described above. Twenty four hours post transfection, cells were changed to assay media [XF base medium (Cat#103575-100, Agilent ) containing 25 mM glucose, 1 mM sodium pyruvate and 1 mM L-glutamine, pH 7.4] and kept in a 37 °C in an incubator without CO_2_ for 60 min. Oxygen consumption rate (OCR) was recorded when cells were metabolically perturbed by the sequential injections of oligomycin (1.5μM), carbonyl cyanide-4-(trifluoromethoxy) phenylhydrazone (FCCP) (2μM) and Rotenone/antimycin A (0.5μM) final concentration). From the obtained OCR values calculations were made as follows: basal respiration (third basal measurement), ATP-linked (difference between basal respiration rate and oligomycin-induced respiration), proton leak (difference between oligomycin-induced and antimycin A-induced respirations), maximal respiration (maximum rate after FCCP injection), reserve capacity (maximal respiration–basal respiration) and non-mitochondrial respiration.

Live-Cell Imaging Using Photoactivatable GFP and LAMP1/Mt-Mscarlet Reporters

AML12 cells were cultured on either 14mm or 20mm coverslips in a 35mm dish (Mattek) that were either tissue culture treated or coated with 1mg/ml poly-d-lysine (PDL; Sigma) at a 1:20 dilution. Cells were transfected using JetPrime (PolyPlus) with 1µg of total DNA and 2µl of transfection reagent in 100µl of buffer per dish/well to be transfected. For MLKL overexpression, 500ng of DDK tagged MLKL (pCAG MLKL-DDK) was transfected alongside 50ng of the fluorescent reporter being used for each experiment (pCAG 2xmt-paGFP p2a 2xmt-mScarlet, pCAG 2xmt-mScarlett or pCAG Lamp1-mEmerald) and the additional 450ng was made up with a pLKO empty vector (ThermoFisher). Following transfection, media was swapped with fresh media to remove transfection reagent after 4-6 hours of incubation at 37 degrees Celsius.

Following overexpression, live time lapse imaging of the cells was done every 15 seconds for 10 minutes on a Nikon Ti2 widefield system equipped with a Hammamatsu ORCA-Fusion CMOS camera, a custom penta-band cube for 378/474/554/635/735 excitation with an Aura III light engine, and 60x (1.4NA) oil objective, and live imaging chamber from OXO (UNO-T-H-CO2) with objective warmer. In addition, a 405nm laser (LUN-F, 50mW) with XY galvo control (Opti-microscan) is connected to perform targeted ROI based stimulation. The whole system is controlled by Nikon Elements. For photo-activation experiments, 5µm by 5µm ROIs were selected in each cell imaged. A before stimulation image was taken, then each box was individually stimulated at 2% laser power for 100µs followed immediately by the time series imaging. For analysis of mitochondrial dynamics, a square ROI of 5µm by 5µm was used and ROI intensity statistics were quantified across the time course. Data was plotted over the entire time course, with comparisons made with end points and slopes.

Exosome Isolation and Nanoparticle Tracking Analysis

AML12 cells were transfected with either control pcDNA or pcDNA-MLKL-Flag as described in the Methodology section. HepG2 cells (HB-8065, ATCC) were transfected with either siControl (Silencer Select Negative Control No. 1 siRNA, 4390843, Thermofisher) or siMLKL (Silencer Select siRNA against human MLKL, #4392420, Thermofisher), and 6 h post transfection, cell culture medium was changed into exosome free medium 24 h before harvest. Exosome isolation was performed using the Total exosome isolation kit (4478359, Invitrogen) as per manufacturer’s instruction. Briefly, collected cell culture media was centrifuged at 2000 x g for 30min. The supernatant was mixed with the total exosome isolation reagent and incubated overnight at 4 °C. After centrifugation at 10,000 x g for 60min at 4°C, exosomes were contained in the pellet at the bottom of the tube. The pellet was then resuspended in PBS, and analyzed using Nanosight (NTA-NS300, Malvern Panalytical) for particle size and concentration analysis. The quantification was done with GraphPad Prism, and the exosome concentration is presented as fold change to the control group.

Necroptosis induction in RAW 264.7 cells

RAW 264.7 cells (TIB-71, ATCC, VA, USA) were polarized to M1 phenotype by treating with 100ng/ml lipopolysaccharide (LPS, L2654, Sigma-Aldrich) for 16 h. The following day, fresh media was added and the cells were treated with 30μM Nec-1s (S8641, Selleck Chemicals, TX, USA) which is a specific RIPK1 inhibitor or with 1 μM GSK-872 (HY-101872, MedChem Express, NJ, USA) which inhibits RIPK3. Corresponding vehicle control was maintained. After 30 minutes of pre-treatment, TBZ (10ng/ml TNFα 410-MT-010/CF, R&D Systems), 5 μM BV6 (533965, EMD Millipore, MO, USA), 20 μM ZVAD-fmk (V116, Sigma-Aldrich) cocktail was added and treated for 24 h.

Study Design and Characteristics of Young and Old Human Cohorts for Plasma Analysis

The study was conducted in the Translational Geroscience Laboratory of the Center for Geroscience and Healthy Brain Aging at the University of Oklahoma Health Sciences Campus. The cognitive status and medical history of the candidate participants were screened to determine eligibility prior to participation. Inclusion criteria consisted of the ability to read and write in English, adequate hearing and visual acuity necessary for the examinations and competence to provide informed consent. Individuals with any of the following conditions were excluded: dementia (Mini Mental State Examination Score < 21), uncontrolled hypertension, uncontrolled diabetes mellitus, history of cerebrovascular or significant cardiac disease (e.g., heart failure), multiple sclerosis, chronic obstructive pulmonary disease, active cancer, established vascular disease, clinically significant anemia (<10 g dL−1). Participants refrained from consuming caffeinated beverages or any substances affecting the alertness for at least 6 hours prior to physiological assessments. All participants were enrolled in the study after obtaining signed informed consent. All procedures and protocols were approved by the Institutional Review Board of the University of Oklahoma Health Sciences Campus (No. 9555). Blood was collected from participants in two, 40 mL K2 EDTA Plus Blood Collection Tubes (BD Vacutainer). Blood was centrifuged at 2500 relative centrifugal force for 15 min. Platelet‐poor plasma samples were stored at −80°C.

Human plasma MLKL quantification

MLKL quantification was performed using MLKL ELISA Kit (#MBS9300811, MyBioSource). Informed consent was obtained from all subjects involved in the study involving metabolic dysfunction-associated steatohepatitis (MASH) patients. Study involving young and aged human cohorts was done in collaboration with the Translational Geroscience Laboratory of the Center for Geroscience and Healthy Brain Aging at the University of Oklahoma Health Campus. All participants were enrolled in the study after obtaining signed informed consent. All procedures and protocols were approved by the Institutional Review Board of the University of Oklahoma Health Campus (No. 9555).

Measurement of mitochondrial respiration and oxidative stress

Mitochondrial respiration in intact cells was measured using Agilent Seahorse XF96 Extracellular Flux Analyzer (Agilent, CA, USA). AML12 cells were transfected with empty vector (pcDNA) or MLKL-Flag and treated with vehicle (EtOH: H2O 1:1) or 250nM of Mitoquinol (MitoQ) (29317, Cayman Chemical Company). MitoQ treatment was initiated 6 h post-transfection and maintained for 24 h prior to analysis (18 h of MitoQ treatment). Mitochondrial respiration was assessed using the Seahorse Mito Stress Test. For assessing oxidative stress, 5μM MitoSox Red staining was done as per manufacturer's instruction (M36008, Thermofisher scientific, MA, USA) and imaged with Nikon Ti Eclipse inverted microscope.

Bioinformatics

For lipid metabolites, significantly altered concentrations between experimental conditions were identified using specific functions provided by the Bioconductor package limma (http://www.bioconductor.org/packages/release/bioc/html/limma.html). Moderated t-statistics, relying on empirical Bayes shrinkage of the standard errors toward a common value, were computed using the lmfit function of the package. The P-values corresponding to the moderated t-statistics were adjusted for multiple testing by false discovery rates (FDR) method of Benjamini, Hochberg, and Yekutieli. FDR below 0.05 was used as filtering criteria for significantly altered metabolites. Families of metabolites significantly enriched in altered metabolites were further identified using Fisher test.


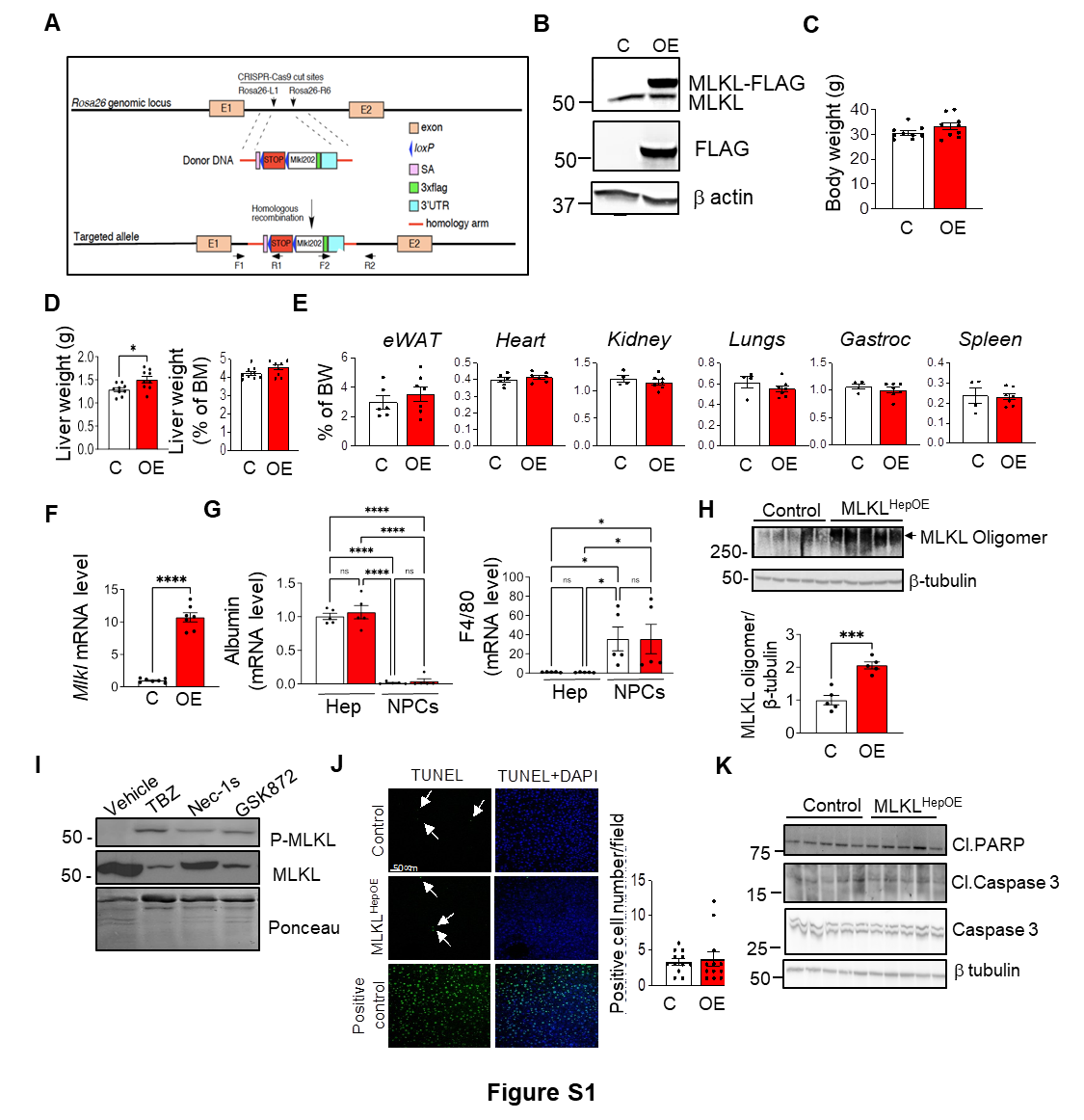


Figure S1**. (A)** Schematic representation of the Mlkl genomic locus and the strategy used to generate MLKL^HepOE^ mice. Exons are depicted as boxes, with loxP sites flanking exon 2 shown as blue triangles. To enable conditional overexpression, a donor DNA construct encoding a stop codon followed by the *Mlkl*-3xFlag sequence was inserted into the *Rosa26* locus via CRISPR-Cas9–mediated homologous recombination. **(B)** Western blot analysis of liver tissue lysates from control and MLKL^HepOE^ mice showing MLKL overexpression, 2 weeks post virus injection. β-actin serves as a loading control. (C) Body weight comparison between control and MLKL^HepOE^ mice. **(D)** Absolute liver weight (g) and liver-to-body weight ratio (% of body mass) in control and MLKL^HepOE^ mice (E) Weights of epididymal white adipose tissue (eWAT), heart, kidney, lungs, gastrocnemius muscle (Gastroc), and spleen, expressed as a percentage of body weight (% BW), in control and MLKL^HepOE^ mice. (F) qPCR analysis of *Mlkl* mRNA levels in the livers of control and MLKL^HepOE^ mice. (G) qPCR analysis of *albumin* and *F4/80* levels in isolated hepatocytes (Hep) and non-parenchymal cells (NPCs) isolated from control and MLKL^HepOE^ mice. **(H)** *Top*: Western blot analysis showing MLKL oligomers in liver tissue lysates from control and MLK^HepOE^ mice. *Bottom*: Quantification of MLKL oligomers normalized to β-tubulin. (I) RAW264.7 cells were polarized to M1 phenotype by LPS treatment, and treated with vehicle, RIPK1 inhibitor necrostatin 1s (Nec-1s, 30μM) or RIPK3 inhibitor GSK872 (1μM) for 30 minutes prior to the addition of TBZ for 24 h. Protein expression of p-MLKL and MLKL were checked by western blotting, β tubulin is used as loading control. (J) *Left:* Representative images of TUNEL staining (green) with DAPI counterstaining (blue) in liver sections from control, MLKL^HepOE^ mice, and a positive control (DNase treated liver sections). Arrows indicate TUNEL-positive nuclei. ***Right:*** Quantification of TUNEL-positive cells per field (4 fields/section; n = 3 animals per group). (K) Western blot analysis of cleaved PARP (Cl. PARP), cleaved caspase 3 (Cl. Caspase 3), and total caspase 3 protein levels in liver lysates of control and MLKL^HepOE^ mice. β-tubulin serves as loading control. Control (white) and MLKL^HepOE^ (red). Data are presented as mean ± SEM from n=7-9mice/group. Statistical significance was determined by two-tailed unpaired t-test for C-F, H or One-way ANOVA for G . ****p < 0.0001, ***p<0.001, *p < 0.05, ns: not significant.


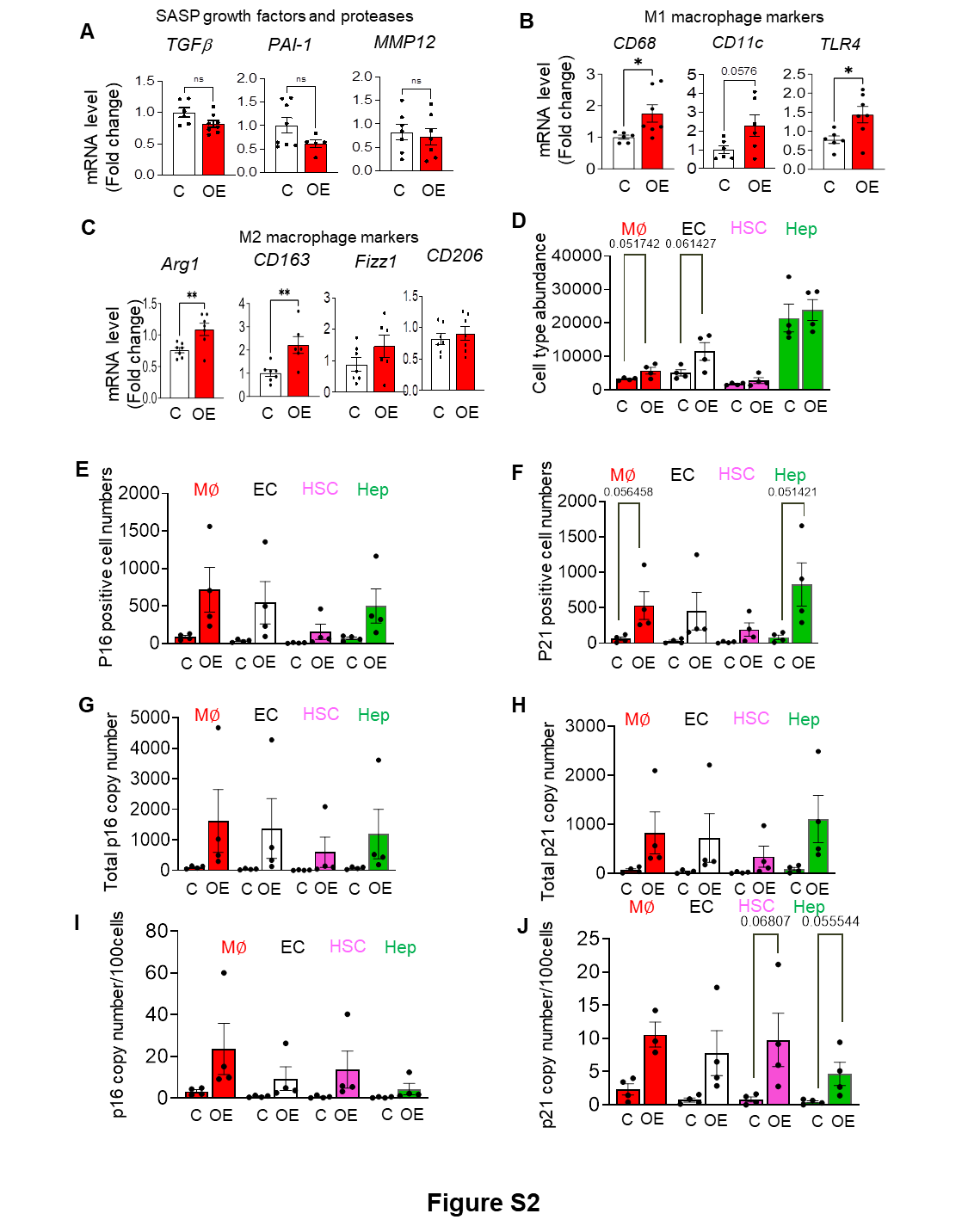


**Figure S2. Data from control and** MLKL^HepOE^ **mice livers. qPCR analysis of (A) SASP factors. (B) M1 macrophage markers (C) M2 macrophage markers. (D) Cell type abundance (absolute) -Total number of a certain cell type within each group shown to represent the cell type abundance. Absolute p16/p21 double-positive counts (E) p16 or (F) p21 positive cell numbers within a certain cell type is shown. Total (G) p16 or (H) p21 copy numbers in a certain cell type. (I) p16 or (J) p21 copy numbers within 100 cells in each cell type. For A-C:** Control (C, white) and MLKL^HepOE^ (OE, red). For C-H: Red bars represent macrophages, white bars represent endothelial cells, pink bars represent hepatic stellate cells and green bars represent hepatocytes. Data are presented as mean ± SEM from n=7-9mice/group (A-C); n=4 mice/group (D-H). Statistical significance was determined by two-tailed unpaired t-test. ****p < 0.0001, ***p<0.001, *p < 0.05, ns: not significant.

**
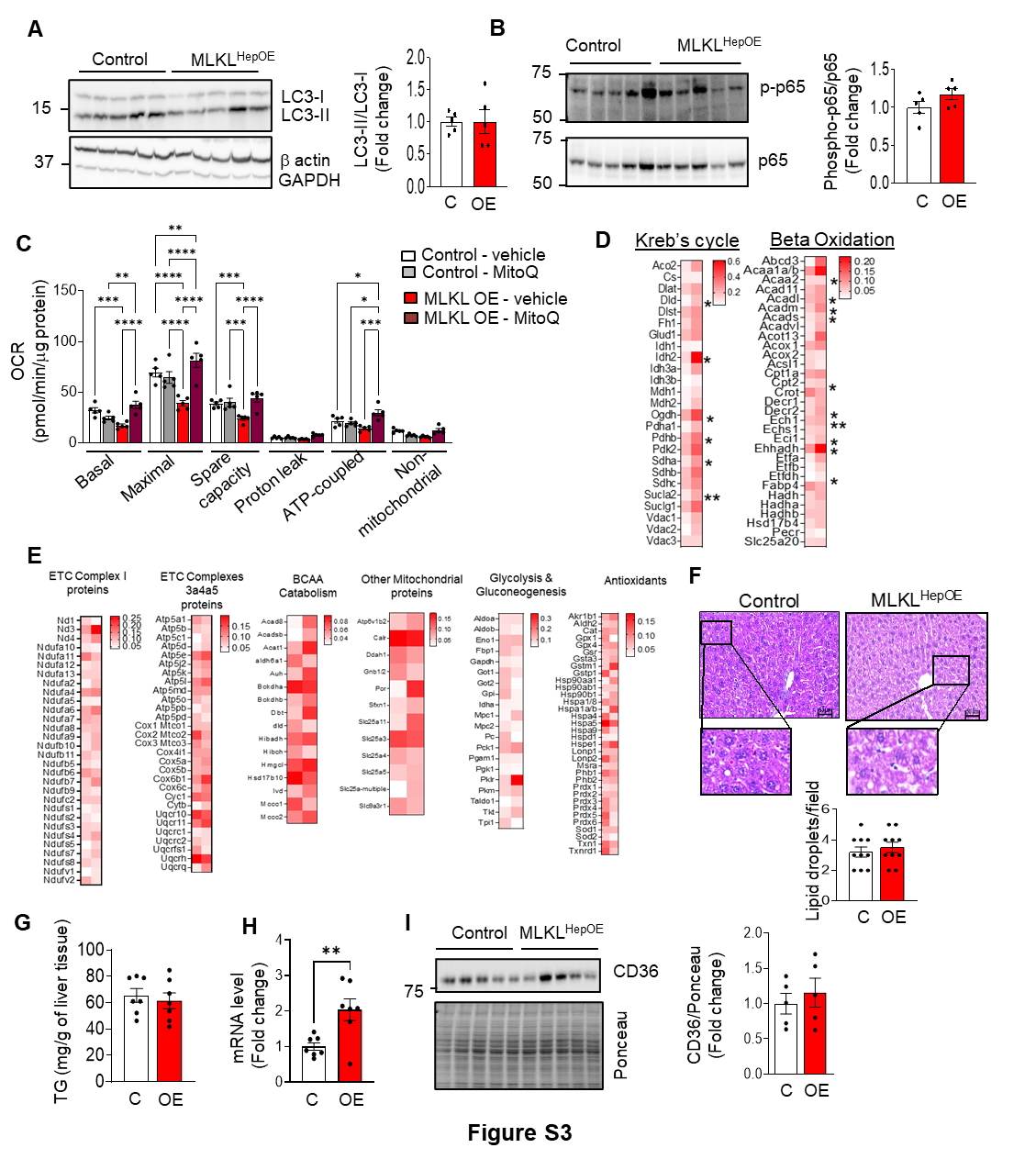
**

Figure S3. Western blot analysis of liver tissue lysates from control (C, white) and MLKL^HepOE^ (OE, red) mice showing **(A) *Left:*** LC3-I, LC3-II and β-actin (loading control), *Right*: Ratio of LC3-II/LC3-I represented as fold change, **(B)** *Left:* Phosphorylated p65 (Phospho-p65), total p65, *Right:* Phospho-p65/p65 ratio, presented as fold change. **(C)** AML12 cells were transfected with empty vector (pcDNA) or MLKL-Flag and treated with vehicle [EtOH: H2O (1:1)] or MitoQ (250 nM). MitoQ treatment was initiated 6 h post-transfection and maintained for 24 h prior to analysis. Mitochondrial respiration was assessed using the Seahorse Mito Stress Test. (D) Heat maps of proteins involved in Kreb’s and TCA cycle obtained by targeted mitochondrial proteomics of liver tissues from MLKLHepOE vs control mice. Red represents up-regulation and white denotes no significant change. Asterisks (*) denote statistically significant changes between the two groups. (E) Heatmap of differentially expressed proteins in the mitochondrial pathways in the livers of MLKL^HepOE^ mice compared to control mice, as determined by targeted proteomics. **(F) Representative images of H&E-stained liver sections from short term (5.5 months) MLKL^HepOE^ mice. Scale bar=50 μm. Magnification: 100X. The graph on the bottom shows quantification of lipid droplets per field (n = 3-4 fields per animal, 3 animals per group) (G) Triglyceride (TG) content (mg/g) of liver tissue (H-I) qPCR (H) and western blot analysis (I) of CD36 in livers from 5.5-month-old control and MLKL^HepOE^ mice. *Right*: Quantification of CD36 normalized to Ponceau staining.** Data from control (C, white) and MLKL^HepOE^ (OE, red) mice (n = 5-7/group). Statistical significance was determined by two-tailed unpaired t-test. **p<0.01, ns: not significant. All data are presented as mean ± SEM from n=5mice/group**.** Statistical significance was determined by two-tailed unpaired t-test, except C (Two-way ANOVA).

**
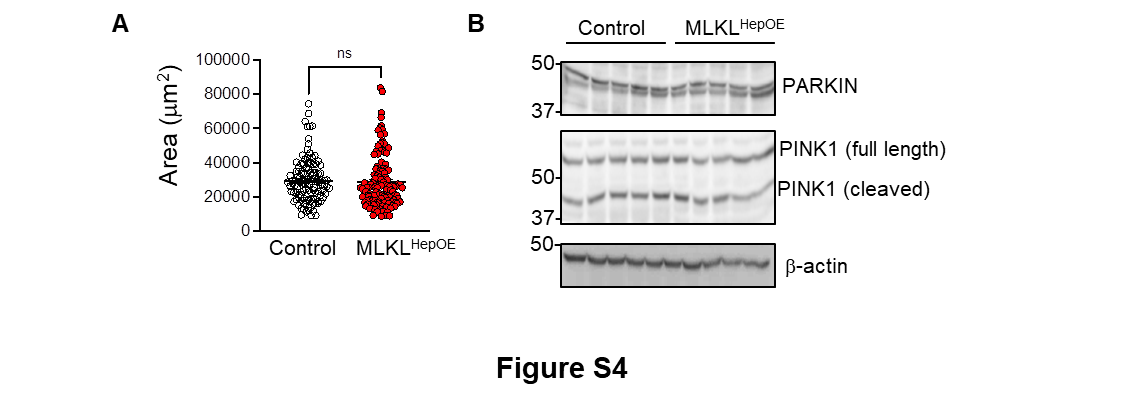
**

Figure S4**. (A) Mitochondrial area (µm²) in liver sections of control (black circles) and MLKL^HepOE^ (red dots) mice. Each dot represents an individual mitochondrion. (B) Western blot analysis showing protein levels of PARKIN, full-length PINK1, and cleaved PINK1 in liver lysates from control and MLKL^HepOE^ mice. β-actin** serves as a loading control. All data are presented as mean ± SEM from n=5mice/group**.** Statistical significance was determined by two-tailed unpaired t-test ns: not significant.


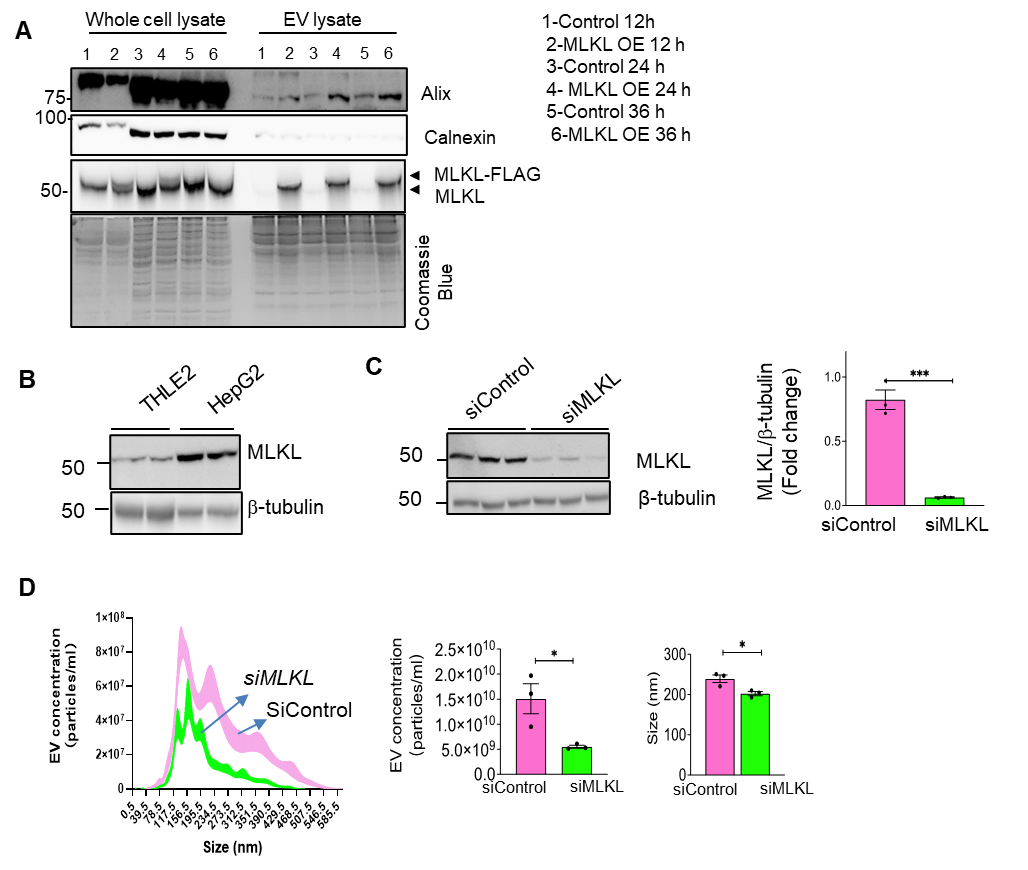


Figure S5. (A) AML12 cells were transfected with either empty vector (pcDNA, control) or pcDNA-MLKL-FLAG (MLKL-OE) for 12, 24 or 36 h. The cell culture supernatant was used for isolating EVs. Lysates were prepared from the cells and EVs, and western blot analysis was performed for EV marker (Alix), ER marker (Calnexin) and MLKL. Coomassie blue stained gel is used as loading control. (B) Western blot analysis of basal MLKL protein expression in THLE2 (normal) and HepG2 (liver cancer) cell lines. β-tubulin is used as a loading control. (C) *Left:* Western blot analysis of MLKL protein expression in HepG2 cells transfected with either siControl or siMLKL. β-tubulin serves as loading control. *Right:* The bar graph shows quantification of MLKL protein levels normalized to β-tubulin, presented as fold change (n=3 independent experiments). (D) Nanoparticle Tracking Analysis (NTA) of EVs isolated from HepG2 cells transfected with siControl (pink) or siMLKL (green). The left panel shows the EV size distribution, the middle panel presents total EV concentration (particles/ml), and the right panel displays average EV diameter (nm) (n = 3 independent experiments). Statistical significance was determined by two-tailed unpaired t-test. ***p < 0.001, *p<0.05.

Table S1: List of primer sequences used for quantitative RT PCR analysis

Table S2: Details of patient samples used for human plasma MLKL analysis in MASH patients

Table S3: Details of samples used for human plasma MLKL analysis in young and old cohort

Table S4: List of significantly altered proteins in untargeted label free quantitative proteomic analysis

Table S5: List of proteins identified by targeted mitochondrial proteomics

Table S6: List of differentially regulated lipid species in lipidomic analysis
