# Supplementary material for: A Non‐Canonical Role for Hepatocyte MLKL in Promoting Mitochondrial Dysfunction and Senescence in the Aging Liver
Source: Aging Cell. 2026 Jul 3;25(7):e70618. doi: 10.1111/acel.70618 (PMC13331751; doi:10.1111/acel.70618)
Supplement: Supplementary file 2 — Table S1: List of primer sequences used for quantitative RT PCR analysis. [file ACEL-25-e70618-s005.docx]

**Table S1: Details of primers used in the study**

| **Primer name** | **Forward primer sequence** | **Reverse primer sequence** |
| --- | --- | --- |
| Mlkl | 5'-CTGAGGGAACTGCTGGATAGAG-3' | 5'-CGAGGAAACTGGAGCTGCTGAT-3' |
| Albumin | 5’-GCGCAGATGACAGGGCGGAA-3’ | 5’-GTGCCGTAGCATGCGGGAGG-3’ |
| Arg1 | 5'-CAAGACAGGGCTCCTTTCAG-3' | 5'-AAGCAAGCCAAGGTTAAAGC-3' |
| CD11c | 5’-CTGGATAGCCTTTCTTCTGCTG-3’ | 5’-GCACACTGTGTCCGAACTC-3’ |
| CD163 | 5’- GGCTAGACGAAGTCATCTGCAC-3’ | 5’-CTTCGTTGGTCAGCCTCAGAGA-3’ |
| CD206 | 5’-ACTACACACTCATCCATTACAACCAA-3’ | 5'-GGCACCTATCACAATCAGGAGGA-3' |
| CD36 | 5’- GGACATTGAGATTCTTTTCCTCTG -3’ | 5’- GCAAAGGCATTGGCTGGAAGAAC-3’ |
| CD68 | 5’-CCACAGGCAGCACAGTGGAC-3’ | 5’-TCCACAGCAGAAGCTTTGGCCC-3’ |
| CXCL1 | 5’- ACCGAAGTCATAGCCACACTC-3’ | 5’- CTCCGTTACTTGGGGACACC-3’ |
| CXCL2 | 5’-CCTGGTTCAGAAAATCATCCA-3’ | 5’-CTTCCGTTGAGGGACAGC-3’ |
| CXCL10 | 5’- ATCATCCCTGCGAGCCTATCCT-3’ | 5’- GACCTTTTTTGGCTAAACGCTTTC-3’ |
| F4/80 | 5'-CCCCAGTGTCCTTACAGAGTG-3' | 5'-GTGCCCAGAGTGGATGTCT-3' |
| Fizz1 | 5'-AGGAACTTCTTGCCAATCCA-3' | 5'-ACAAGCACACCCAGTAGCAG-3' |
| IFN gamma | 5'-CTTCTTCAGCAACAGCAAGGCG-3' | 5'-ATGCTTGGCGCTGGACCTGTG-3' |
| IL1β | 5’-AGGTCAAAGGTTTGGAAGCA-3’ | 5’-TGAAGCAGCTATGGCAACTG-3’ |
| MMP12 | 5’- TGCACTCTGCTGAAAGGAGTCT-3’ | 5’- GTCATTGGAATTCTGTCCTTTCCA-3’ |
| p15 | 5'-ATCCCAACGCCCTGAACCGCT-3' | 5'-AGTTGGGTTCTGCTCCGTGGAG-3' |
| p16 | 5’-CCCAACGCCCCGAACT-3’ | 5’-GCAGAAGAGCTGCTACGTGAA-3’ |
| p21 | 5’-GTCAGGCTGGTCTGCCTCCG-3’ | 5’-CGGTCCCGTGGACAGTGAGCAG-3’ |
| p53 | 5’-GTATTTCACCCTCAAGATCC-3’ | 5’-TGGGCATCCTTTAACTCTA-3’ |
| Pai-1 | 5′-GACACCCTCAGCATGTTCATC-3′ | 5′-AGGGTTGCACTAAACATGTCAG-3′ |
| Ripk3 | 5'-GAAGACACGGCACTCCTTGGTA-3' | 5'-CTTGAGGCAGTAGTTCTTGGTGG-3' |
| TGFβ | 5’-ACCATGCCAACTTCTGTCTGGGAC-3’ | 5’-ACAACTGCTCCACCTTGGGCTTG-3’ |
| TLR4 | 5'-ATGGCATGGCTTACACCACC-3' | 5'-GAGGCCAATTTTGTCTCCACA-3' |
| TNFα | 5’-CACAGAAAGCATGATCCGCGACGT-3 | 5’- CGGCAGAGAGGAGGTTGACTTTCT-3’ |

**Table S1**: List of primer sequences used for quantitative real time PCR analysis
