# Supplementary material for: A Non‐Canonical Role for Hepatocyte MLKL in Promoting Mitochondrial Dysfunction and Senescence in the Aging Liver
Source: Aging Cell. 2026 Jul 3;25(7):e70618. doi: 10.1111/acel.70618 (PMC13331751; doi:10.1111/acel.70618)
Supplement: Supplementary file 4 — Table S3: Details of samples used for human plasma MLKL analysis in young and old cohort. [file ACEL-25-e70618-s006.docx]

**Table S3: Details of samples used for human plasma MLKL analysis from young and old cohorts**

| **Sample number** | **Tube ID** | **Age at visit** | **BMI** | **Sex** |
| --- | --- | --- | --- | --- |
| 1 | CN084 | 31 | 21.5 | F |
| 2 | CN129 | 33 | 18.0 | F |
| 3 | CN034 | 51 | 28.5 | F |
| 4 | CN086 | 56 | 24.3 | F |
| 5 | CN085 | 58 | 33.3 | F |
| 6 | CN103 | 60 | 37.8 | F |
| 7 | CN131 | 61 | 25.8 | F |
| 8 | CN114 | 63 | 30.6 | F |
| 9 | CN108 | 64 | 17.4 | F |
| 10 | CN116 | 66 | 23.3 | F |
| 11 | CN095 | 67 | 30.0 | F |
| 12 | CN012 NEW | 68 | 23.7 | F |
| 13 | CN110 | 70 | 19.9 | F |
| 14 | CN102 | 71 | 29.5 | F |
| 15 | CN112 | 74 | 24.6 | F |
| 16 | CN098 | 78 | 27.3 | F |
| 17 | CN106 | 81 | 25.8 | F |
| 18 | CN119 | 81 | 27.5 | F |
| 19 | CN005 | 25 | 23.9 | M |
| 20 | CN121 | 25 | 23.9 | M |
| 21 | CN120 | 30 | 20.8 | M |
| 22 | CN130 | 33 | 23.8 | M |
| 23 | CN082 | 38 | 24.1 | M |
| 24 | CN030 | 41 | 20.5 | M |
| 25 | CN124 | 41 | 25.2 | M |
| 26 | CN067 | 42 | 26.8 | M |
| 27 | CN104 | 42 | 26.1 | M |
| 28 | CN089 | 49 | 20.7 | M |
| 29 | CN123 | 54 | 28.0 | M |
| 30 | CN094 | 58 | 30.0 | M |
| 31 | CN127 | 59 | 25.7 | M |
| 32 | CN128 | 65 | 27.6 | M |
| 33 | CN096 | 66 | 20.0 | M |
| 34 | CN009 NEW | 67 | 26.1 | M |
| 35 | CN078 | 68 | 34.2 | M |
| 36 | CN100 | 73 | 25.1 | M |
| 37 | CN118 | 75 | 31.0 | M |
| 38 | CN047 | 76 | 34.8 | M |
| 39 | CN113 | 76 | 29.0 | M |
| 40 | CN016 NEW | 81 | 33.5 | M |
| 41 | CN109 | 82 | 20.8 | M |
| 42 | CN097 | 86 | 25.8 | M |
| 43 | CN107 | 87 | 27.4 | M |

M - Male; F - Female
